# Evolutionary Patterns of the Genes Involved in the Integrity and Segregation of Chromosomes in Sawflies (Hymenoptera: Symphyta)

**DOI:** 10.1002/ece3.73748

**Published:** 2026-06-01

**Authors:** Ayşe Rümeysa Nalça, Ertan Mahir Korkmaz

**Affiliations:** ^1^ Department of Molecular Biology and Genetics, Faculty of Science Sivas Cumhuriyet University Sivas Turkey

**Keywords:** gene duplication, purifying selection, sawflies, structural maintenance of chromosomes

## Abstract

The genes involved in the regulation of the meiotic process act important roles in maintaining the structural integrity of chromosomes, ensuring accurate mitotic segregation, and preserving genome stability. Although the structural and functional components of the cohesin and condensin complexes have been extensively investigated in model organisms, their evolutionary diversification and conservation levels in insects remain largely unknown. Here, we characterized and comparatively analyzed the architectural and functional features of the genes encoding these complexes in the more primitive and largely phytophagous suborder Symphyta (Hymenoptera: Insecta). The genome and transcriptome datasets representing 11 sawfly families and six superfamilies of sawflies were analyzed to identify the genes. These analyses identified 10 and 12 genes, as well as a total of 46 conserved motifs. The physicochemical properties and amino acid composition of these proteins are consistent with nucleoplasmic functions, acting main roles in DNA binding and protein–protein interactions. The result of phylogenetic analyses provides evidence for the hypothesis that the *SMC* genes originated from an ancient duplication of a single ancestral *SMC* gene. The absence of the *CAPG2* gene in certain species, coupled with the occurrence of species‐specific duplication events in most of the genes, indicates substantial heterogeneity in the evolutionary dynamics of these complexes. Despite the strong purifying selection pressure acting on the genes, positive selection signals were predicted in certain positions of the *CAPG2*, *RAD21*, and *SA* genes. These findings suggest that these genes exhibit conserved and species‐specific evolutionary features in this insect lineage, consistent with their central roles in chromosome organization and cell cycle regulation.

## Graphical abstract

This study identifies and characterizes the genes encoding cohesin and condensin complexes across Symphytan superfamilies, revealing their structural and functional roles with conserved motifs in maintaining genome stability. Phylogenetic analyses indicate that *SMC* genes originated from ancient duplication events, with notable gene loss (e.g., *CAPG2*) and species‐specific duplications shaping their evolution. Despite strong purifying selection, localized positive selection in certain genes suggests both conserved and lineage‐specific evolutionary dynamics.

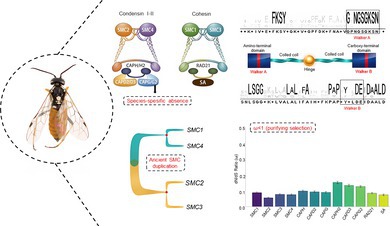

## 1 Introduction

Cell division and chromosome dynamics are highly crucial biological processes ensuring the viability of eukaryotic organisms. The genes involved in the regulation of the meiotic process represent specialized and evolutionarily conserved multi‐subunit structures retaining the structural integrity and accurate segregation of chromosomes (Hagstrom and Meyer 2003; Hirano 2016). Structural Maintenance of Chromosomes (SMC) proteins play a vital role in the organization of the genome and its subsequent expression during this process. These structurally conserved proteins also regulate chromosomal DNA from bacteria to humans (Hirano 2005). They are multi‐protein complexes that utilize the energy derived from ATP hydrolysis to facilitate the organization of chromosomes (Hoencamp and Rowland 2023). They also interact with diverse proteins to form different complexes. Since SMC complexes share evolutionarily conserved structural similarities, they also organize DNA using similar mechanisms (Yuen and Gerton 2018). In the majority of eukaryotes, there are at least six genes (*SMC1‐6*) encoding SMC proteins that are structurally conserved yet perform distinct cellular functions (Harvey et al. 2002). The cohesin complex, which plays a role in sister chromatid cohesion and gene regulation, contains the SMC1 and SMC3 proteins (Golov and Gavrilov 2024). In contrast, the condensin complex, responsible for chromosome condensation, includes the SMC2‐SMC4 heterodimer (Jessberger 2003; Pandey et al. 2020). Additionally, the SMC5‐SMC6 heterodimer is part of the complex involved in DNA recombination and repair (Hoencamp and Rowland 2023; Schleiffer et al. 2003).

In contrast, many bacteria and archaea possess a single SMC homolog that forms a homodimer. SMC proteins, ranging in size from approximately 110 to 170 kDa, consist of an NH2‐terminal nucleotide triphosphate‐binding domain, two long coiled‐coil regions (~50 nm) separated by a hinge, and a COOH‐terminal domain (Skibbens 2019). The long coiled‐coil region is highly conserved among bacterial and eukaryotic SMC proteins (Hirano 2016; Krepel et al. 2018; Uhlmann 2016).

SMC complexes rely on ATP hydrolysis to interact with chromosomes (Yoshinaga and Inagaki 2021). The N‐ and C‐terminal regions of each SMC protein form “head” domains with ATPase activity, capable of binding and hydrolysing two ATP molecules (White and Erickson 2009). These domains associate with kleisin subunits (e.g., CAPH/H2, RAD21) to create ring‐shaped complexes. Additionally, HEAT (Huntingtin, elongation factor 3, the A subunit of protein phosphatase 2A, TOR lipid kinase) subunits interact with kleisins to assemble condensin and cohesin complexes (King et al. 2019). SMC proteins belong to the ABC (ATP binding cassette) ATPase superfamily, sharing conserved ATPase domains, including N‐terminal Walker A (P‐loop) and C‐terminal Walker B motifs, as well as six specific sequence motifs: A, R, Q, D, H‐loop, and the C‐motif (signature motif) (Hudson et al. 2008; Neuwald and Hirano 2000; Palou et al. 2018).

Condensins are conserved five‐subunit protein complexes involved in mitotic chromosome condensation and segregation. They are first identified proteins in studies using *Xenopus laevis* egg extracts (Hudson et al. 2008; Kimura and Hirano 1997). Subsequent research has identified two highly conserved condensin complexes, condensin I and condensin II, in most eukaryotes (Hirano 2016; Hirota et al. 2004). Condensin I is localized in the cytoplasm and interacts with chromosomes only after nuclear envelope breakdown during prometaphase, contributing to lateral chromosome compaction. In contrast, condensin II resides in the nucleus during interphase and facilitates the axial compaction of chromosomes (Cobbe and Heck 2006; Hirano 2016; Wallace et al. 2015). Eukaryotic condensin complexes share the same heterodimeric SMC2 and SMC4 subunit pair, along with three non‐SMC subunits. These non‐SMC subunits consist of a kleisin (CAPH/H2) and a pair of HEAT subunits (CAPD2/D3, CAPG/G2) (Anderson et al. 2002; White and Erickson 2009; Wood et al. 2010). Condensin I encompasses CAPH, CAPD2, and CAPG, whereas condensin II contains CAPH2, CAPD3, and CAPG2 (Cobbe and Heck 2004; Hirano 2012; Hirota et al. 2004; Hudson et al. 2008).

Cohesins are ring‐shaped protein complexes essential for sister chromatid cohesion during mitotic and meiotic cell division (Hirano 2016; Nasmyth 2011). They play key roles in DNA damage repair and gene expression regulation in post‐mitotic cells (Peters et al. 2008). The function of the cohesin complex begins in the synthesis phase (S phase) and ends with the separation of sister chromatids during anaphase (Samejima et al. 2025; Tvedte et al. 2017). The subunit organization of the cohesin complex is similar to that of the condensin complex (Hirano 2016; Rankin 2015). The mitotic cohesin complex consists of four subunits: two SMC proteins (SMC1/SMC3), a kleisin (RAD21/Rec8), and a HEAT subunit, stromal antigen (SA) (Dorsett 2019; Pherson et al. 2019; Zhang et al. 2013).

Although the structural and functional components of these complexes have been extensively investigated in model organisms, the evolutionary diversification, conservation levels, and functional adaptations of these genes in insect lineages remain largely unknown. In this context, the order of Hymenoptera with its high lifestyle diversity may provide an ideal system for evolutionary biology and genomic studies. The basal suborder Symphyta (sawflies), representing an early‐diverging lineage with less derived genetic traits (Peters et al. 2017), is particularly valuable for comparative analyses of the evolution of cellular regulatory systems.

This study provides a comprehensive and comparative analysis of the architectural and functional characteristics of genes encoding condensin I, condensin II, and cohesin complexes in sawflies (Symphyta), which is the more primitive and largely phytophagous suborder of Hymenoptera. Drawing on the published genome and transcriptome datasets and newly sequenced genome dataset of *Xiphydria prolongata* representing the superfamily of Xiphydrioidea, homologous sequences of the core subunits within these complexes were delineated, with a subsequent rigorous examination of their structural features, motif formations, and conserved domain architectures and duplication patterns. Furthermore, intragenomic and intergenomic (comparisons at the family/superfamily level) evolutionary dynamics of these genes in the sawfly species were revealed through phylogenetic inference and selective pressure analyses, unveiling key adaptive insights.

## 2 Results

### 2.1 Genome/Transcriptome Assembly and Annotation

The first genome assembly of the willow wood borer *Xiphydria prolongata* was generated from short‐read (150 bp) Illumina sequencing with approximately 80× coverage (Table 1). The assembly consisted of 22.903 scaffolds (> 1 kb) with a total length of 277 Mb. Details of the read quality and sequencing datasets for all sampled sawfly species, including the firstly sequenced species, are presented in Table 1. The Q30 values varied between 81.51% (*Xyela alpigena*) and 98.31% (*Arge pagana*). The estimated genome sizes ranged from 143.5 Mb in *Syrista parreyssii* to 311.9 Mb in *Xiphydria prolongata*. The sequencing coverage of the assembled genomes ranged from 64× in *Athalia japonica* to 786× in *Corynis zhengi*. The basic statistics of the assembled genomes were given in Table S2, ranging from 407 to 22.903 scaffolds, longest transcript of 9.715–26.078, N50 length of 0.531–2.089 Mb and 36%–47% GC content. BUSCO completeness versus the hymenoptera_odb10 reference dataset (*n* = 5.991) was 44.6%–96.9% complete, 0.07%–24.87% duplicated, 0.67%–10.27% fragmented and 2.42%–45.35% missing BUSCOs (Table S3). The number of the predicted protein coding sequences exhibited variation due to the nature of the available dataset, ranging from 9.715 (*Pergagrapta polita*) and 15.818 (*Orussus abietinus*) to 21.576 (*Xyela alpigena*) and 46.891 (*Corynis zhengi*) for the transcriptome and genome datasets, respectively (Table S4).

**TABLE 1:** Read numbers and sequencing quality metrics for the symphytan species.

Superfamily	Family	Species	Read number	Q20 (%)	Q30 (%)	GC (%)	Genome size (bp)	Coverage
Xyeloidea	Xyelidae	*Xyela alpigena*	7.714.749	91.55	81.51	44	—	—
Tenthredinoidea	Pergidae	*Pergagrapta polita*	10.401.403	93.63	84.21	44	—	—
	Argidae	*Arge pagana*	2.317.271	99.07	97.89	43	166.962.108	122×
	Athaliidae	*Athalia japonica*	19.345.813	98.37	95.49	41	180.018.213	64×
	Cimbicidae	*Corynis zhengi*	192.165.186	97.56	93.51	39	146.544.608	786×
	Tenthredinidae	*Tenthredo notha*	1.433.973	98.25	95.92	37	197.351.147	94×
	Diprionidae	*Neodiprion lecontei*	81.874.705	95.32	86.93	40	234.221.782	209×
Pamphilioidea	Pamphiliidae	*Cephalcia chuxiongica*	25.225.337	98.43	94.95	36	—	—
Xiphydrioidea	Xiphydriidae	*Xiphydria prolongata*	84.133.156	96.95	91.22	40	311.856.686	80×
Cephoidea	Cephidae	*Syrista parreyssii*	72.191.158	97.86	93.83	41	143.482.581	301×
Orussoidea	Orussidae	*Orussus abietinus*	32.809.057	92.89	84.38	47	177.269.285	74×

### 2.2 Identification of the Genes in the Complexes

A total of 91 putative genes associated with condensin complexes and 51 putative genes associated with cohesin complexes were identified in the genomes and/or transcriptomes of sawfly species (Table S5). Compared to genome datasets, gene members of these complexes were often predicted as partial sequences in transcriptome datasets, as they frequently lacked complete open reading frames (ORFs), including missing 5′ and/or 3′ ends. This may reflect limitations in transcriptome assembly quality or sequencing coverage. The analyses also did not predict homologous sequences of *CAPG2* gene (a component of the condensin II complex) in Cephoidea (*Syrista parreyssii*) and Tenthredinoidea (*Corynis zhengi*, *Neodiprion lecontei*, *Tenthredo notha*, and *Athalia japonica*). Furthermore, the assembly of transcriptome for the species *Cephalcia chuxiongica* (Pamphiloidea) did not encompass the homologous sequences of the *CAPH2* and *CAPD3* genes. Finally, a total of 125 genes were identified as being putatively functional genes and associated with 12 genes representing the condensin (81 genes) and cohesin (44 genes) complexes (Table S5). These genes possessed conserved domains found in these complexes: SMC_N (IPR003395) and SMC_hinge (IPR003395) domains in *SMC* genes; the Cnd2 (IPR022816) domain in *CAPH*; the Cnd1 (IPR032682) domain in *CAPD2* and *CAPD3*; the Cnd3 (IPR025977) domain in *CAPG*; the Condensin2nSMC (IPR031737) domain in *CAPG2*; the CNDH2_C (IPR031737) domain in *CAPH2*; the Rad21_Rec8 (IPR049589) and Rad21_Rec8_N (IPR006910) domains in *RAD21*; and the Scc3/SA (IPR039662) domain in *SA* (Table S6).

### 2.3 Characterization of the Genes in the Complexes

**FIGURE 1:**
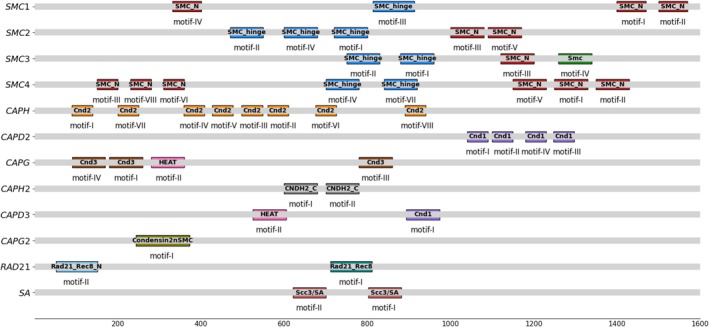
Domain organization of the genes encoding condensin I–II and cohesin complexes in Symphyta. X‐axis shows the amino acid positions of the related genes. Each functional domain is shown in different colors. The consensus amino acid sequence and length of each putative motif associated with a functional protein domain found in the condensin and cohesin complexes are presented in Table S6.

**FIGURE 2:**
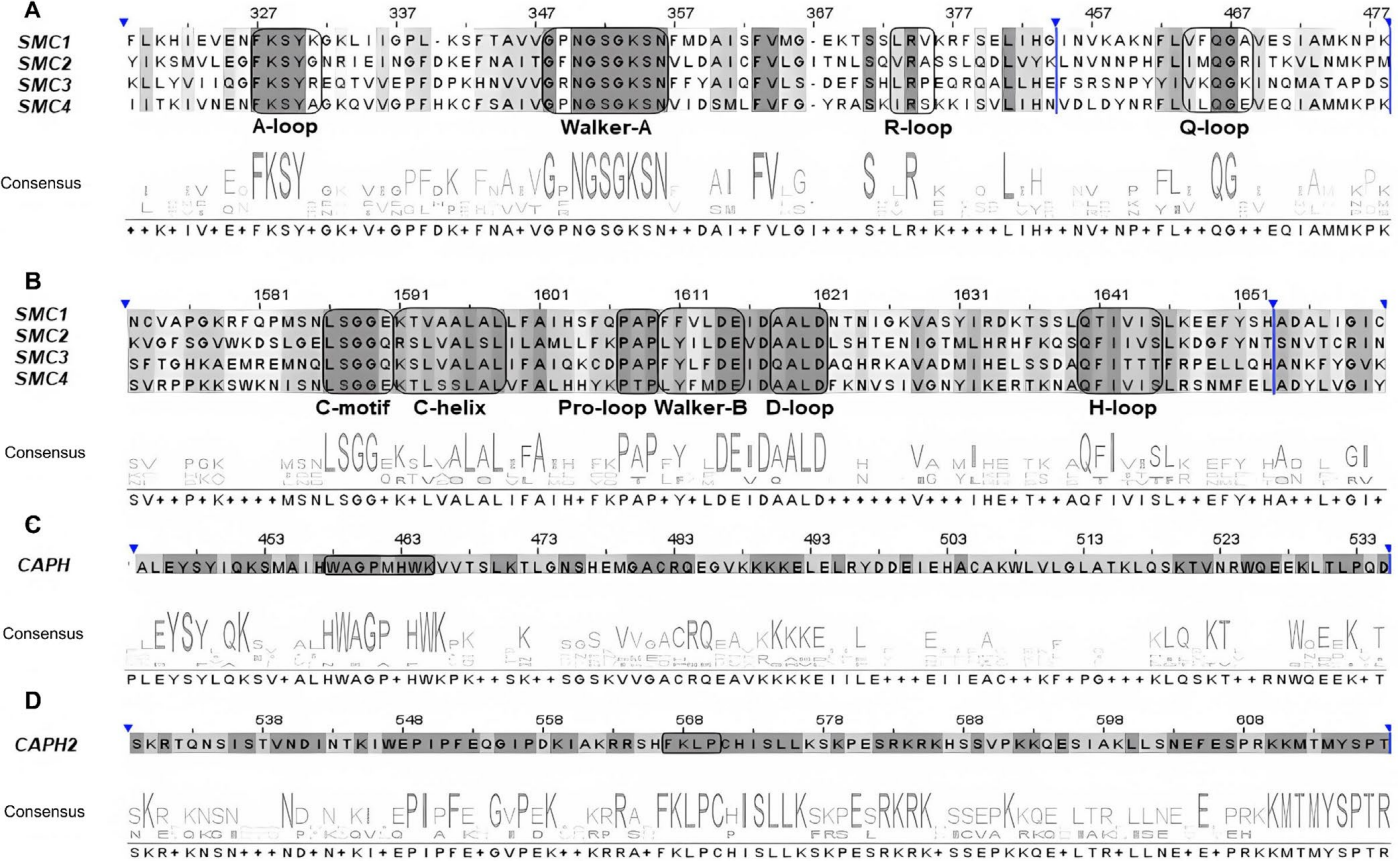
Motif organization of *SMC* (1–4) and *CAPH*/*CAPH2* genes. (A) The A‐loop, Walker‐A, R‐loop, and Q‐loop motifs correspond to the N‐terminal ATP‐binding domain of SMC proteins. (B) The C‐motif, C‐helix, Pro‐loop, Walker‐B, D‐loop, and H‐loop motifs correspond to the C‐terminal ATP hydrolysis domain of SMC proteins. The consensus line indicates the degree of conservation within the motifs. (C) The *CAPH* gene contains the “WA/SGP/AH/FW” motif (in single‐letter amino acid code), which represents part of a unique DNA‐binding region known as the *“safety‐belt”* domain. (D) The *CAPH2* gene contains the Mrg15 nuclear‐binding motif (FKLP; in single‐letter amino acid code), functioning as an important auxiliary factor of the complex.

A total of 46 motifs were found, ranging from one in *CAPG2* to eight in *SMC4* and *CAPH*, varying according to the involved gene (Table S6). The length of these conserved motifs ranged from 21 to 50 amino acids. Furthermore, the motifs in *SMC* and *CAPH* genes were mostly highly conserved, with only one or two variable residues compared to other genes in the complexes. Each of these motifs was related in the known functional protein domains (Figure 1). The motifs predicted in *SMC1*‐*SMC4* genes were located in SMC_N, SMC_hinge, and Smc domains. High sequence conservation was observed particularly in motifs corresponding to the N‐terminal and C‐terminal regions of these *SMC1‐4* genes. The motifs present in the N‐terminal region have been identified as the A‐loop, P‐loop (Walker‐A), R‐loop, and Q‐loop, whilst the motifs found in the C‐terminal region include the C‐motif, C‐helix, Pro‐loop, Walker‐B, D‐loop, and H‐loop (Figure 2A,B). The motifs found in the Kleisin subunits (*CAPH*, *CAPH2*) were observed in the Cnd2 and CNDH2_C domains, while the motifs in the HEAT subunits (*CAPD2*, *CAPD3*, *CAPG*, *CAPG2*) were located within Cnd1, Cnd3, HEAT, and Condensin2nSMC domains (Table S6). The *CAPH* gene encoding the Kleisin subunit of the condensin I complex comprises the “WA/SGP/AH/FW” motif (in the single‐letter amino acid code), which represents a part of the unique DNA‐binding domain termed the “safety belt”. Similarly, the *CAPH2* gene encoding the Kleisin subunit of the condensin II complex includes the Mrg15 nuclear binding motif (FKLP; in the single‐letter amino acid code) as an important co‐factor of the complex. This motif was found to be highly conserved in all analyzed sawfly species (Figure 2C,D). Finally, the motifs in the *RAD21* and *SA* genes of the cohesin subunit were observed to be localized in Rad21_Rec8_N, Rad21_Rec8, and Scc3/SA domains.

The physicochemical properties were estimated for the genes encoding these protein complexes (Table S7). However, as most transcriptome‐derived sequences represent partial genes lacking complete ORFs, only results obtained from genome dataset were used for comparative interpretation. The amino acid length of the condensin I–II complex genes exhibited variability, with a range from 607 (*CAPH2*) to 2943 (*SMC2*), while the cohesin complex varied from 753 (*RAD21*) to 2018 (*SA*). The molecular weights of the protein complex genes varied between 17.85 kDa in *CAPG* of *Orussus abietinus* and 329.03 kDa in *SMC2* of *Corynis zhengi*. The isoelectric points (pI) varied from acidic (4.72 in *RAD21* of *Neodiprion lecontei* ) to basic (9.75 in *CAPH* of *Corynis zhengi*). Furthermore, the majority of the genes were unstable, with instability indices ranging from 25.9 (*Orussus abietinus*, *CAPG*) to 63.7 (*Orussus abietinus*, *RAD21*). GRAVY values displayed that most proteins are hydrophilic, with the exception of *CAPG2* gene in *Arge pagana*.

A comparable nucleotide composition was observed among the complexes, with an average of 56% A+T and 44% G+C content (Figure S1B). The amino acid compositions also exhibit a broadly similar patterns across the three complexes, with relatively high abundance of certain amino acids (Figure S1A). The most abundant amino acids were leucine (Leu, 10.36%), glutamate (Glu, 9.63%), lysine (Lys, 8.77%) and alanine (Ala, 6.87%), accounting for an average of 35.6% of the total composition. Polar amino acids such as serine (Ser, 6.90%), asparagine (Asn, 4.83%) and glutamine (Gln, 5.13%) were also present at relatively high proportions across all complexes. In contrast, the tryptophan (Trp, 0.49%), cysteine (Cys, 1.27%), histidine (His, 2.00%) and tyrosine (Tyr, 2.40%) were at lower levels, together accounting for an average of 6.16% of the total composition. A comparison of the RSCU values also suggested no strong codon usage bias among the analysed genes (data not shown).

**FIGURE 3:**
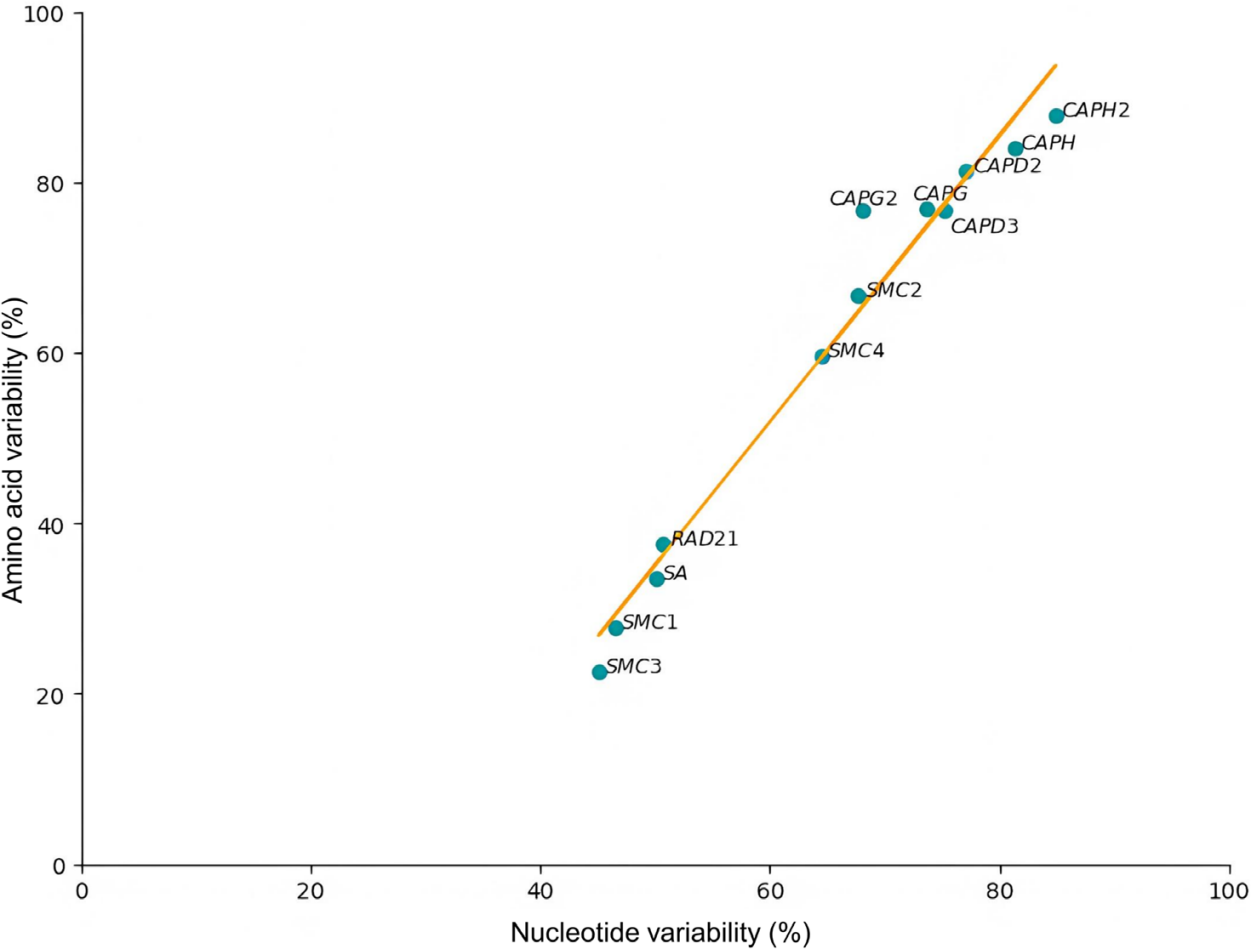
Nucleotide and amino acid sequence variability (%) among condensin I, condensin II, and cohesin complex genes. Each blue point represents an individual gene. Variability values were calculated as the proportion of variable sites relative to the total alignment length based on multiple sequence alignments in MEGA7.

The genes that encode the protein complexes diverge noticeably between sawfly species (Figure 3). Similarities for each gene were in low levels ranging from 54.9% (nt div.) and 77.4% (aa div.) in *SMC3* to 15.1% (nt div.) and 12.1% (aa div.) in the *CAPH* gene. Within the condensin I complex, nucleotide variability ranged from 64.5% to 81.3% and amino acid variability from 59.7% to 83.9%. Notably, the *CAPH2* gene exhibited the highest variability at both the nucleotide (81.3%) and amino acid (83.9%) levels. In contrast, *SMC4* exhibited the least variability, with an average of nucleotide and amino acid variability of 64.5% and 59.7%, respectively. The condensin II complex genes also showed high levels of variability. The *CAPH* gene was found to be particularly variable, with values of 84.9% at the nucleotide and 87.9% at the amino acid level. However, the genes of the cohesin complex exhibited markedly lower variability (Figure 3). For instance, *SMC1* and *SMC3* displayed nucleotide variability of 46.5% and 45.1%, respectively, while their amino acid variability was notably lower at 27.7% and 22.6%.

### 2.4 Phylogenetic Relationship and Duplication Dynamics of the Complexes

**FIGURE 4:**
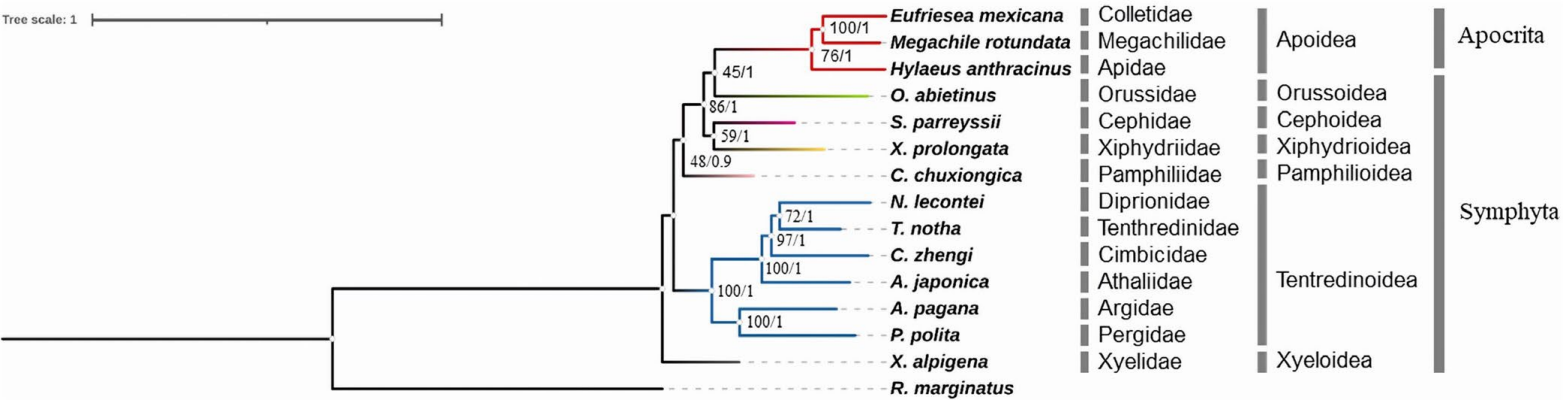
Species tree of the suborder Symphyta based on the concatenated amino acid sequences of condensin I–II and cohesin genes. Phylogenetic trees were reconstructed using Maximum Likelihood (ML) and Bayesian Inference (BI) methods. Bootstrap support values (ML) and posterior probabilities (BI) are shown above the corresponding nodes, respectively. *Ricinus marginatus* was used as an outgroup.

**FIGURE 5:**
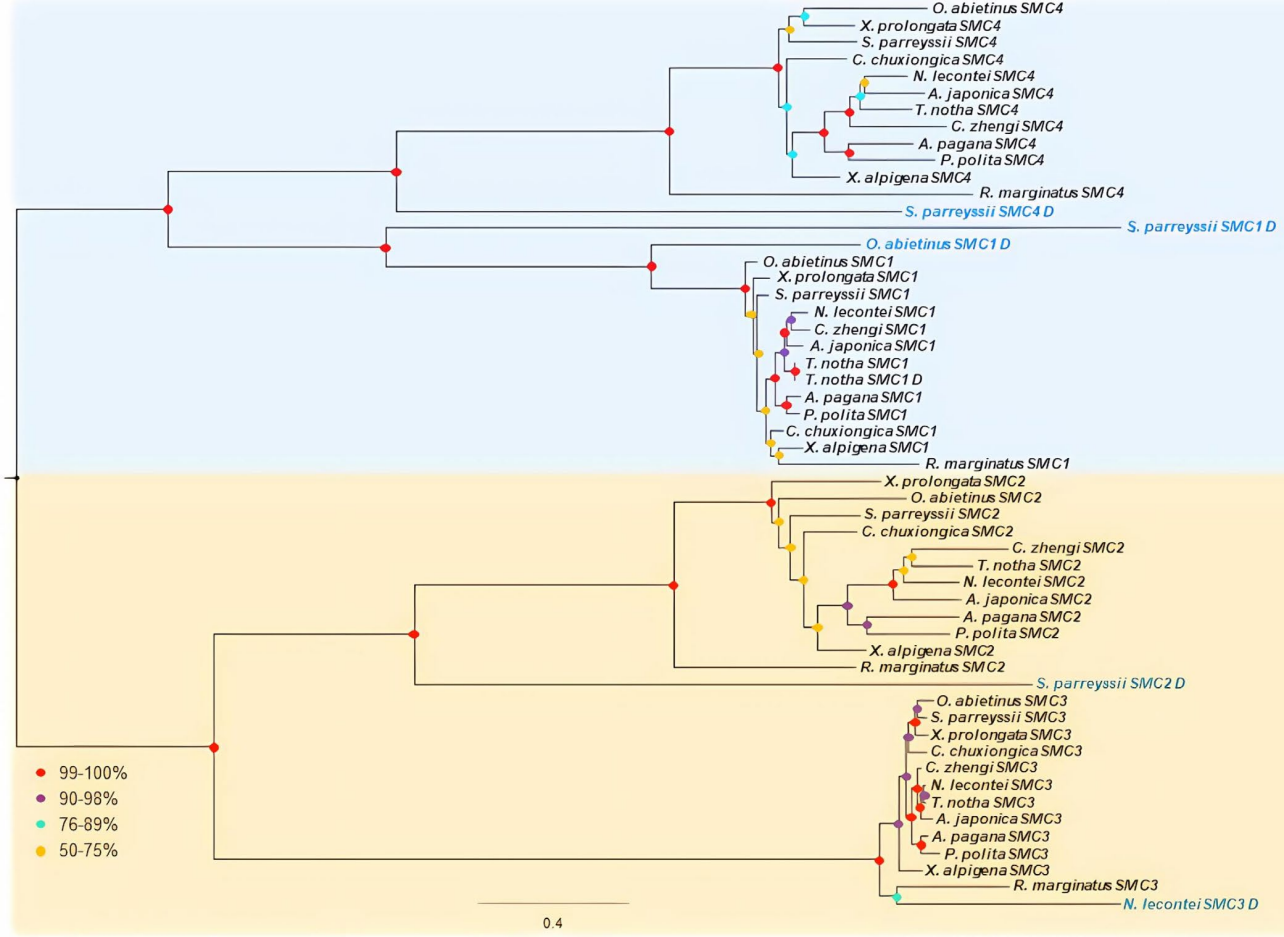
The recovered gene tree from the concatenated amino acid sequences of *SMC* genes using the Maximum Likelihood (ML) method. Bootstrap support values are indicated at the nodes. *Ricinus marginatus* was used as an outgroup.

**FIGURE 6:**
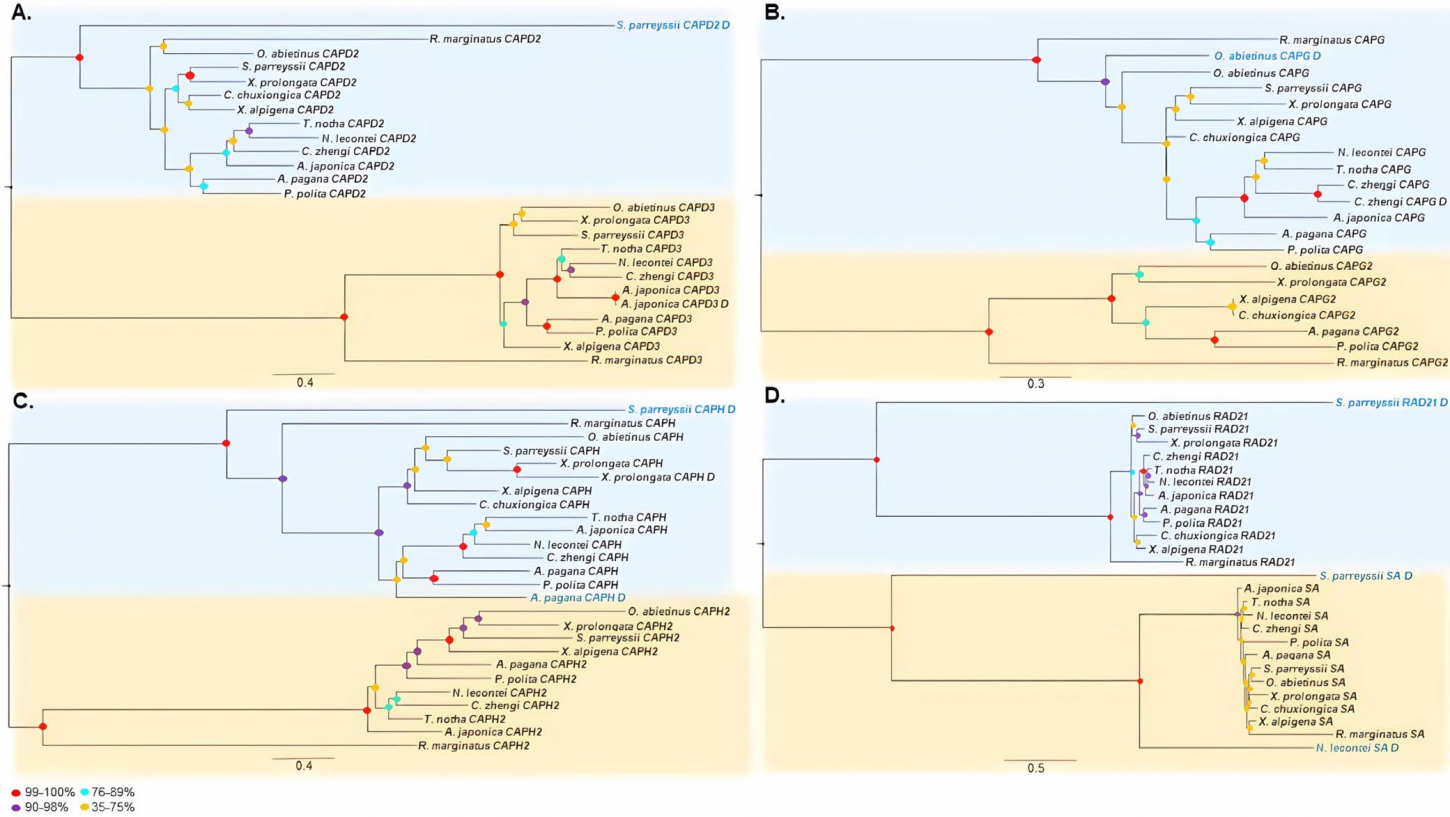
The gene trees representing of the concatenated aminoacid sequences of (A) *CAPD2*/*CAPD3*, (B) *CAPG*/*CAPG2*, (C) *CAPH*/*CAPH2*, (D) *RAD21*/*SA* genes using the Maximum Likelihood (ML) method. Bootstrap support values are indicated at the nodes. The tree is rooted with *Ricinus marginatus* as an outgroup.

The ML and BI trees recovered from the amino acid dataset comprising 12 genes are mostly similar topology and supported the broadly accepted internal phylogeny among the sawfly superfamilies, with high support values (ML bootstrap ≥ 90%; Bayesian posterior probability ≥ 0.90) (Figure 4). The only difference between two inference approaches is the placement of the superfamily of Pamphilioidea, which formed a sister group with Xyeloidea in the BI tree. Each SMC homolog formed a distinct, well‐supported group in the phylogeny of the *SMC* gene homologs (Figure 5). A sister group relationship has been recovered between SMC1 and SMC4, and SMC2 and SMC3 lineages, supporting the hypothesis that these lineages emerged through ancestral duplication events in early eukaryotic evolution (Beasley et al. 2002; Schurko et al. 2009; van Hooff et al. 2024; Yoshida et al. 2022). In addition, each SMC homolog appears to have evolved largely vertically across species. *SMC* gene homologs were mostly present as single copies in the genomes of sawflies, with the exception of the *SMC1* (*Syrista parreyssi*, *Orussus abietinus* and *Tenthredo notha*), *SMC2* (*Syrista parreyssii*), *SMC3* ( *Neodiprion lecontei* ) and *SMC4* (*Syrista parreyssii*), which appear to have undergone independent duplication events with two copies. Phylogenetic analyses of the HEAT subunit genes (*CAPD2* and *CAPD3*; *CAPG* and *CAPG2*) suggested that these genes are predominantly present as single copies, although sporadic duplication events were observed in *CAPD2* (*Syrista parreyssii*) (Figure 6A), *CAPD3* (*Athalia japonica*) (Figure 6A), and *CAPG* (*Orussus abietinus* and *Corynis zhengi*) (Figure 6B). Similarly, the kleisin subunit genes (*CAPH* and *CAPH2*) were largely single‐copy, with the exception of *CAPH* duplications detected in *Syrista parreyssii*, *Arge pagana*, and *Xiphydria prolongata* (Figure 6C). Within the cohesin complex, *RAD21* and *SA* genes were mostly single copy, except for duplications in *RAD21* homologs (*Syrista parreyssii*) and *SA* homologs (*Syrista parreyssii* and *Neodiprion lecontei* ) (Figure 6D). Notably, duplicated gene copies in SMC, HEAT, kleisin, and cohesin subunits tended to exhibit longer branch lengths compared to single‐copy genes near the base of each homologous group in the phylogenetic trees. Excluding *SMC1_D* (*Tenthredo notha*) (Figure 5), *CAPD3_D* (*Athalia japonica*) (Figure 6A), *CAPG_D* (*Corynis zhengi*) (Figure 6B), and *CAPH_D* (*Xiphydria prolongata*) (Figure 6C), which likely resulted from more recent duplications, these divergent copies may represent remnants of ancient duplication events that have subsequently been lost across sawfly lineages.

### 2.5 Gene‐Level Selection Analyses

**FIGURE 7:**
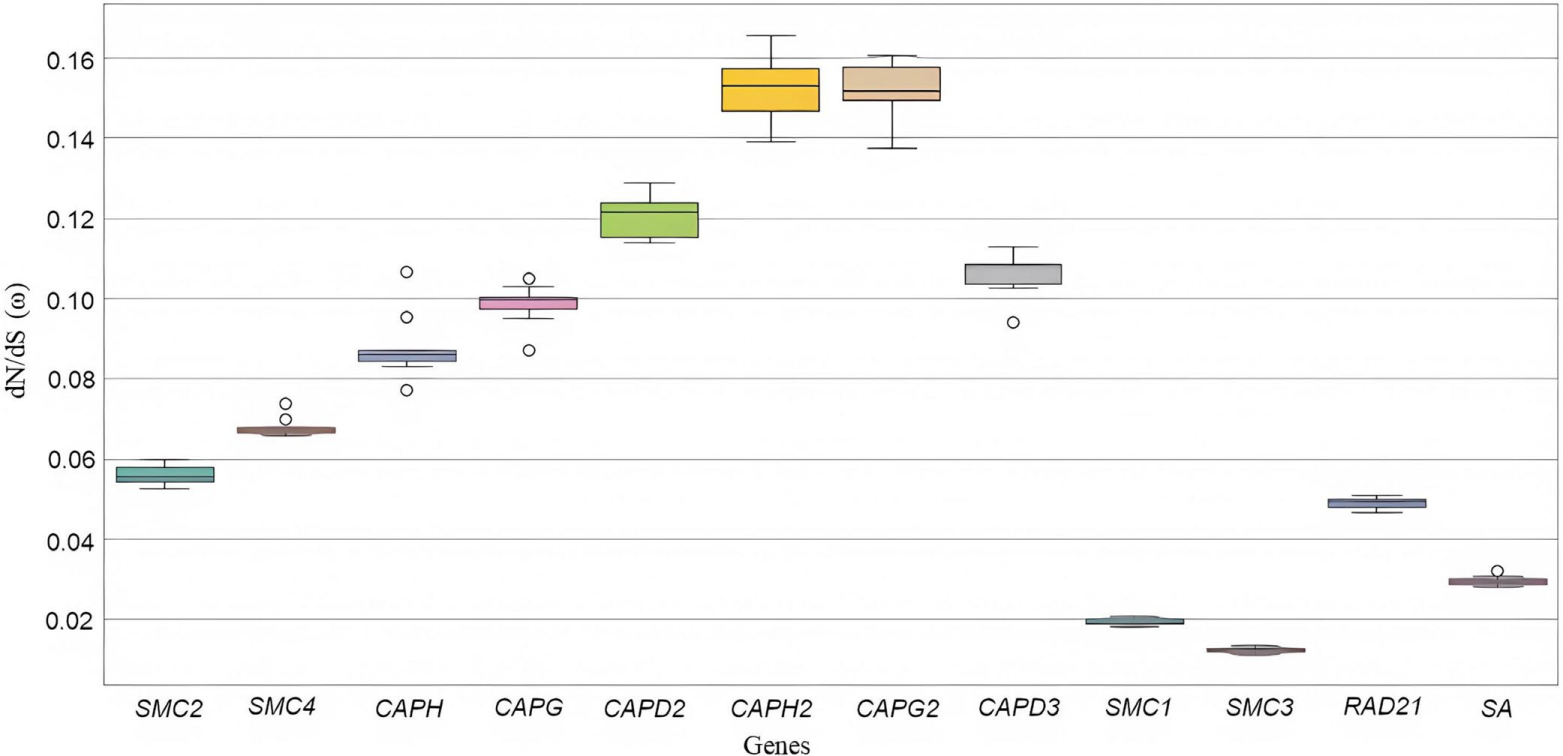
Gene and species‐specific dN/dS (*ω*) ratios of condensin I, condensin II, and cohesin complex genes among the symphytan species.

**FIGURE 8:**
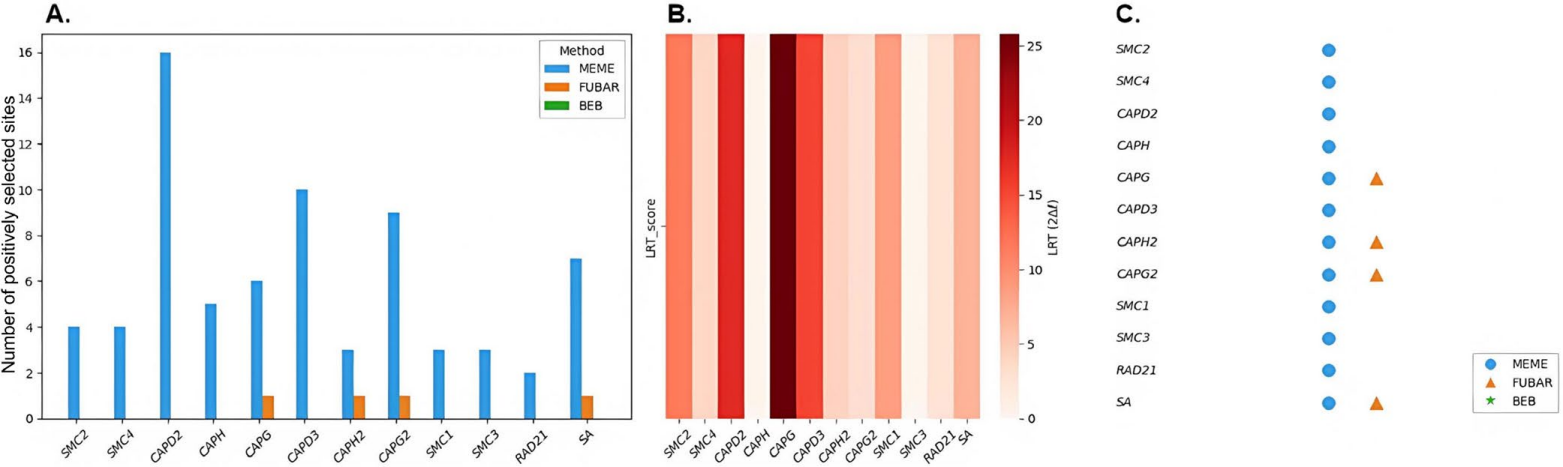
Comparison of the results of positive selection analysis based on the genes and preferred approaches. (A) The number of codons detected under positive selection for each gene. (B) Likelihood Ratio Test (LRT) scores as a heat map (Higher values indicate a stronger positive selection signal). (C) The preferred method (shown with symbols) for positive selection of each gene.

The evolutionary analysis of these genes across sawflies provides insights into their selection pressures. Using the branch model, *ω* (dN/dS) ratios ranged from 0.0560 (*SMC2*) to 0.0985 (*CAPG*) for condensin I, 0.0560 (*SMC2*) to 0.1528 (*CAPH2*) for condensin II, and 0.0122 (*SMC3*) to 0.0489 (RAD21) for cohesin, all with *ω* < 1 (Figure 7; Table S9), indicating strong purifying selection and high conservation due to their essential cellular roles. Site model analyses (M8 vs. M7) suggested potential positive selection in *SMC1*, *SMC2*, *SMC4*, *CAPD2*, *CAPG*, *CAPD3*, *CAPH2*, and *SA* genes (LRT > 3.841), but no codon positions reached significant posterior probability (≥ 0.95) in the BEB analysis, displaying weak evidence for codon‐level positive selection (Table S10). Branch‐site model analyses detected positive selection signals in specific lineages, but certain positions were not statistically significant in LRT comparisons (Table S11), and identified codon positions did not overlap with conserved SMC motifs. Further analyses using HyPhy's FUBAR and MEME methods indicated the presence of both pervasive and episodic positive selection signals (Table S10 and Figure 8). MEME detected episodic positive selection signals at several codon positions, including those in condensin I (e.g., *SMC2*: 347, 353, 841, 1165; *CAPD2*: multiple sites), condensin II (e.g., *CAPD3*: 72, 177, 185; *CAPH2*: 214, 228), and cohesin (e.g., *SMC1*: 520, 862; *RAD21*: 344, 688). FUBAR identified fewer pervasive positive selection sites, including *CAPG* (142), *CAPH2* (202), *CAPG2* (412), and *SA* (1182) (Figure 8). The strongest support for positive selection was observed in *CAPG2* (412), *RAD21* (344), and *SA* (1266) (Tables S10 and S11), supported by both MEME and either FUBAR or BEB, suggesting these codon positions may have adaptively evolved to support functional roles in these complexes.

## 3 Discussion

The evolutionary comparison of genes associated with the condensin I–II and cohesin complexes suggests the presence of conserved features alongside structural and selective variations across the sawfly superfamilies. The present study identified functional genes ranging from 10 to 12 across six sawfly superfamilies (Table S5). The observed variation in the number of identified functional genes may be attributable to sporadic duplication events, as has been frequently reported in these complexes (Schurko et al. 2009, 2010; Tvedte et al. 2017). The majority of the identified genes of *Pergagrapta polita*, *Xyela alpigena*, and *Cephalcia chuxiongica* were recovered as partial in the transcriptome datasets. A similar pattern has been reported in other eukaryotic organisms, where certain condensin subunits are underrepresented in transcriptome datasets (Yoshinaga and Inagaki 2021). Although CAPG2 homologs were found at both the genomic and transcriptomic levels in most species, their absence in Cephoidea (*Syrista parreyssii*) and some families of Tenthredinoidea (*Corynis zhengi*, *Neodiprion lecontei* , *Tenthredo notha*, and *Athalia japonica*) is particularly noteworthy. While this pattern may reflect gene loss, species‐specific absence, or limitations in assembly and transcriptome coverage, the evolutionary loss of the *CAPG2* gene is notably supported by reports across several insect orders (King et al. 2019; Johnson et al. 2013; Martin and Wang 2011). This pattern suggests that *CAPG2* may have undergone independent losses during insect evolution. Its absence in these symphytans likely reflects a relaxed selection pressure or functional redundancy, potentially compensated for by other complex components. Supporting this, our failure to recover corresponding sequences from raw sequencing reads confirms their true absence rather than an assembly artefact.

The motifs identified in condensin I–II and cohesin complex genes appear to be evolutionarily conserved, suggesting important roles in maintaining the structural integrity and functional capacity of these proteins. In particular, the N‐terminal (A‐loop, P‐loop/W‐A, R‐loop, Q‐loop) and C‐terminal (C‐motif, Pro‐loop, Walker‐B, D‐loop, H‐loop) motifs observed in SMC1–4 proteins constitute the structural basis for their essential roles in cellular processes (Figure 2A,B). These motifs are highly conserved and primarily related to ATP binding, hydrolysis, and conformational transitions. Notably, the P‐loop (Walker A) motif forms the structural element responsible for binding the phosphate groups of ATP, whereas the Walker B motif facilitates ATP hydrolysis and regulates the opening and closing of the SMC protein ring (Harvey et al. 2002; Hirano 2016; Nasmyth and Haering 2005). The “safety‐belt” motif in the kleisin subunit CAPH and the Mrg15 nuclear‐binding motif in CAPH2 are consistent with the proposed roles in DNA attachment and chromatin interactions (Kinoshita et al. 2022; Wallace et al. 2015) (Figure 2C,D). The localization of motifs within the HEAT subunits (CAPD2, CAPD3, CAPG, and CAPG2) in the Cnd1, Cnd3, and HEAT domains highlights their involvement in chromatin organization and protein–protein interactions. Similarly, the motifs identified in the *RAD21* and *SA* genes, corresponding to the Rad21_Rec8, Rad21_Rec8_N, and Scc3/SA domains, emphasize their crucial regulatory roles in mitotic and meiotic divisions (King et al. 2019) (Table S6). The presence of these motifs across the sawflies species indicates that the mechanical functions of SMC proteins have remained largely conserved throughout their evolutionary history and may be maintained by purifying selection. Moreover, this pattern suggests that the ATPase motor activity controlling the opening and closing of condensin and cohesin rings performs fundamental biological functions in early diverging hymenopteran lineages.

The observed variations in amino acid length, molecular weight, isoelectric point, and stability index can be attributed to the functional diversity and structural heterogeneity of the complexes (Table S7). The extended polypeptide chains and elevated molecular weights of *SMC* genes are consistent with their proposed roles in complex architecture and ring structure regulation via ATPase activity (Hirano 2006). Predominantly negative GRAVY values can be related to hydrophilic, soluble in aqueous environments, and thus well‐adapted for nucleoplasmic functions of these proteins. However, the positive GRAVY value of *CAPG2* in *Arge pagana* indicates relatively higher hydrophobicity, which may correspond to altered subcellular localization or specialized protein–protein interactions. Although hydrophobic residues such as leucine and alanine are frequent, the strong hydrophilicity of charged and polar amino acids, which possess markedly negative hydropathy indices, outweighs their contribution, resulting in overall negative GRAVY values. At the same time, the enrichment of leucine and alanine, together with charged residues such as lysine and glutamate (Figure S1A), may contribute to the formation of internal hydrophobic cores while facilitating electrostatic interactions with DNA, thereby supporting proper protein folding and DNA binding (Dill 1990). Additionally, the abundance of polar amino acids (serine, asparagine, and glutamine) probably enhances protein solubility and interaction potential (Ptak‐Kaczor et al. 2021). Conversely, the low prevalence of cysteine suggests a reduced reliance on disulfide bond–mediated stabilization and cysteine‐targeted post‐translational modifications, while the low abundance of tryptophan may indicate fewer highly specific aromatic interaction interfaces (Boutin et al. 2024; Shao et al. 2022). Overall, the amino acid composition aligns with the roles of cohesin and condensin in chromosome organization and cell cycle regulation. Notably, the relatively high frequency of charged residues, particularly lysine and glutamate (Figure S1A), highlights the potential importance underscores of electrostatic interactions in DNA binding.

Phylogenetic analyses of SMC, HEAT, kleisin, and cohesin subunit genes revealed that these genes have been shaped by both ancestral and species‐specific duplication events (Figures 5 and 6). The *SMC* genes form well‐supported monophyletic clades separating the SMC1/SMC4 and SMC2/SMC3 lineages across the sawfly species (Figure 5). This pattern provides robust evidence for the hypothesis that these pairs originated from an ancient duplication of a single ancestral SMC gene early in eukaryotic evolution (Beasley et al. 2002; Schurko et al. 2009), aligning with current models on the evolutionary origins of eukaryotic condensin and cohesin complexes (van Hooff et al. 2024; Yoshida et al. 2022). The occurrence of most SMC, HEAT, and kleisin homologs as single‐copy genes in many species may reflect their critical functional roles and conservation under purifying selection (Figure 7). However, the presence of multiple copies of SMC complex genes appears to be related to species‐specific independent duplication events, which likely reflect sporadic duplications contributing to the evolutionary diversification of SMC complex genes (Figure 5). The appearance of duplicated copies with the longer branch in the gene trees indicates that these sporadic duplications may have lost their coding potential through rapid accumulation of deleterious mutations (Lynch and Conery 2000). This is also consistent with the pseudogenization, which is the most common fate of gene duplications. Conversely, the presence of duplicated copies with shorter branch lengths (*Tenthredo notha* SMC1_D (Figure 5), *Athalia japonica* CAPD3_D (Figure 6A), *Corynis zhengi* CAPG_D (Figure 6B), and *Xiphydria prolongata* CAPH_D (Figure 6C)) suggests they may have occurred more recently.

Purifying selection (all *ω* (dN/dS) ratios below 1) is predominantly observed in these genes, supporting their main role in fundamental cellular processes such as chromosome segregation, DNA condensation, and sister chromatid cohesion, and that loss‐of‐function mutations are eliminated by natural selection (Figure 7; Table S9). In spite of purifying selection acting on all genes, the detection of positively selected codon sites outside of known functional domains (see Table S10) suggests that the selection is concentrated in regions involved in protein–protein interactions, intracellular localisation or regulatory flexibility rather than in enzymatic or structural cores. These positions, often found in less conserved regions, may have developed adaptive responses to interspecies differences in environmental or physiological pressures.

## 4 Experimental Procedures

### 4.1 Sampling, DNA Extraction and Sequencing

An ethanol‐preserved male specimen of *Xiphydria prolongata* was obtained from the Evolutionary Bioinformatics Research Group Collection, Sivas Cumhuriyet University, Sivas, Türkiye (EBRG). Total genomic DNA was extracted using a standard salting‐out protocol (Sunnucks and Hales 1996) and quantified with the Qubit dsDNA HS Assay Kit on a Qubit 4.0 Fluorometer (Thermo Fisher Scientific, USA). The extracted DNA was pooled and subjected to paired‐end (150 bp) sequencing on the DNBSEQ‐G400 platform (MGI Tech Co. Ltd., Shenzhen, China).

### 4.2 Construction of Datasets and Quality Assessment

A sampling dataset of sawfly insects (Hymenoptera: Symphyta) representing 11 families across six superfamilies was generated, including (i) the newly sequenced genome of one species, and (ii) genome datasets of seven species and transcriptome datasets of three species manually retrieved from the NCBI Sequence Read Archive (SRA). All details of the sampling dataset are provided in Table 2. Each selected SRA file was downloaded and converted to FASTQ format using the “prefetch” and “fasterq‐dump” commands in SRA Toolkit v3.0.1 (https://github.com/ncbi/sra‐tools; accessed 12 December 2024). Raw NGS reads from both genome and transcriptome datasets were assessed using FastQC v0.11.9 (Andrews 2010). Reads containing adaptor contamination, duplicates, low‐quality bases (< Q20), or poly‐N stretches (> 5 bp) were removed using fastp v0.20.0 (Chen et al. 2018). The filtered clean reads were then used to estimate genome size and heterozygosity using Jellyfish v2.2.6 (Manekar and Sathe 2018) and GenomeScope v2.0 (Vurture et al. 2017) with a k‐mer size of 21.

**TABLE 2:** Data sources of the symphytan species used in this study.

Superfamily	Family	Species	SRA/Genome	SRA/Transcriptome	References
Xyeloidea	Xyelidae	*Xyela alpigena*		SRX642930	Peters et al. (2017)
Tenthredinoidea	Pergidae	*Pergagrapta polita*		SRX642961	Peters et al. (2017)
	Argidae	*Arge pagana*	ERX11438828		—
	Athaliidae	*Athalia japonica*	SRX23781575		—
	Cimbicidae	*Corynis zhengi*	SRX9735396		—
	Tenthredinidae	*Tenthredo notha*	ERX6561450		—
	Diprionidae	*Neodiprion lecontei*	SRX981417		—
Pamphilioidea	Pamphiliidae	*Cephalcia chuxiongica*		SRX10368867	
Xiphydrioidea	Xiphydriidae	*Xiphydria prolongata*	SRR35876269		This study
Cephoidea	Cephidae	*Syrista parreyssii*	SRX19719519		—
Orussoidea	Orussidae	*Orussus abietinus*	SRX330915		Funabiki et al. (2023)

### 4.3 Assembly and Annotation

Scaffolds for the genome datasets of eight species and the transcriptome datasets of three species were assembled using the de Bruijn graph‐based SPAdes v3.15.5 (Bankevich et al. 2012) with the options “‐k 55,87,109,121 ‐‐careful ‐‐cov‐cutoff auto” and rnaSPAdes (Bushmanova et al. 2019) with the “‐only‐assembler” option, respectively. The quality of the assembled genome scaffolds and transcriptome sequences was assessed using BUSCO v5.5.0 (Benchmarking Universal Single‐Copy Orthologs) (Waterhouse et al. 2018) with the hymenoptera_odb10 reference dataset (*n* = 5.991). Summary statistics of the assemblies were calculated using QUAST (Gurevich et al. 2013) and rnaQUAST (Bushmanova et al. 2016). Tandem repeats in the assembled genomes were identified and masked using Tandem Repeat Finder (TRF) (Behboudi et al. 2023). A custom library of repetitive elements was generated by scanning the final genome assemblies using RepeatModeler v2.0.5 (Smit and Hubley 2015). The custom library was then used to identify and mask repetitive elements and low‐complexity regions in the assemblies using RepeatMasker v4.1.6 (Smit et al. 2015). Subsequently, protein‐coding sequences were predicted from the repeat‐masked genomes using the ab initio method implemented in Augustus v3.3.3 (Stanke et al. 2006), and the output was saved in GFF format. Amino acid sequences of the annotated genes were extracted from the GFF files using the getAnnoFasta.pl. script in Augustus, and the output was saved in FASTA format. The prediction of protein‐coding sequences from the transciptome datasets was performed by TransDecoder v5.5.0 (https://github.com/TransDecoder/) under the default parameters. The predicted protein‐coding sequences were functionally annotated by searching against the Pfam, Panther, and Superfamily databases using InterProScan5 v65‐97.0 (Hunter et al. 2012). The potential genes involved in condensin and cohesin protein complexes were then manually extracted.

### 4.4 Identification of the Genes in Condensin and Cohesin Protein Complexes

Genes of the condensin and cohesin complexes from 11 species of the suborder Symphyta were identified using a homology‐based approach. Firstly, full‐length amino acid sequences of the genes from *Apis mellifera* and *Drosophila melanogaster* were retrieved from the UniProt and NCBI Gene databases (Table S1). These species were selected because their well‐annotated genomes in public databases provide experimentally validated and curated condensin and cohesin gene sequences, which were used as reference queries for homology‐based gene identification. Secondly, the retrieved gene sequences were combined, and redundant sequences were removed using CD‐HIT with a sequence identity threshold of 0.95. The remaining non‐redundant, high‐confidence sequences were designated as the reference genes for the condensin and cohesin complexes. These reference genes were then used as queries in BLASTP searches against candidate genes of the complexes from 11 Symphyta species (BLAST v2.15.0+, E‐value = 1 × 10^−5^) (Altschul et al. 1990). The structural domains of the candidate genes were further verified by HMMER (version 3.4) searches (Potter et al. 2018). Finally, these verified genes were evaluated using a combination of criteria, including homology, exon–intron structure, and the presence of premature stop codons or frameshifts, with the Pseudogene Pipeline script (https://github.com/ShiuLab/PseudogenePipeline). After the pseudogene search, the remaining genes were classified as functional if they contained a conserved domain along the respective gene, as determined by InterProScan5 v65‐97.0. To further investigate the genes annotated as missing in the genome assemblies, targeted gene recovery analyses were performed on raw sequencing reads using the aTRAM pipeline (Allen et al. 2015).

### 4.5 Characterization of the Genes in the Complexes

To achieve more accurate identification of genes within the complexes, motif searches were performed on each gene using the MEME Suite (Multiple Expectation Maximization for Motif Elicitation) (Bailey et al. 2015). The motif width was set to 6–50 residues, and the maximum number of motifs was limited to 10. The motifs predicted by MEME were subsequently searched against the Pfam and NCBI‐CDD databases using the Motif Search tool (https://www.genome.jp/tools/motif/). The basic nucleotide and amino acid compositions and relative synonymous codon usage (RSCU) values of each identified gene were calculated using the cusp program in EMBOSS v6.6.0. (Rice 2002). Nucleotide and amino acid sequence variability were assessed using MEGA7 based on multiple sequence alignments, both at the individual gene level and using a concatenated dataset comprising all genes. Finally, physicochemical properties, including molecular weight, theoretical isoelectric point (pI), instability index, aliphatic index, and grand average of hydropathicity (GRAVY), were analyzed using the ProtParam tool (Gasteiger et al. 2005).

### 4.6 Phylogenetic Reconstruction

Phylogenetic analyses were used to assess the usefulness of a dataset including 12 gene to reconstruct the phylogeny of sawflies. Additionally, further phylogenetic analyses were conducted to explore the duplication patterns and evolutionary relationships of the genes within each gene group (*SMC*, *CAPD*, *CAPH*, *CAPG*, and *RAD21*‐*SA*). *Ricinus marginatus* (Psocodea) was selected as an outgroup (Soffan et al. 2018). A range of amino acid datasets were created containing both alignment of each gene, each group and concatenation of all genes. The amino acid dataset for each gene was individually aligned using the MAFFT algorithm under the “‐auto” option and then manually edited (Katoh and Standley 2013). In order to obtain the aligned files for each gene group and all genes, the individually aligned amino acid datasets were then combined using SequenceMatrix v1.10 (Vaidya et al. 2011). The optimal partitioning strategy and the optimal evolutionary model of each partition were selected using PartitionFinder v2.1.1 (Lanfear et al. 2017) by applying a “greedy” algorithm based on “unlinked” estimated branch lengths and Bayesian information criterion. Phylogenetic analyses were conducted under the VT + G and BLOSUM62 substitution models. ML (Maximum Likelihood) analyses were performed in IQ‐TREE v2.1.4 (Minh et al. 2020) using default parameters and allowing it to automatically select the evolutionary model. BI (Bayes Inference) analyses were performed using MrBayes v3.2.7a (Ronquist et al. 2012) with two independent runs of ten million generations with four Markov chains, sampling every 1000 generations, under the associated model and unlinked branch lengths of each partition scheme. Stability was assessed using Tracer v1.7 (Rambaut et al. 2018). The first 25% of trees in each run were discarded as burn‐in, and the remaining trees were used to create a consensus BI tree. Finally, the ML and BI trees were visualized with FigTree v1.4.4 (Rambaut 2018).

### 4.7 Signature of Selective Forces

To assess the role of different selective forces in the evolution of each gene in the condensin and cohesin protein complexes, the ratio of nonsynonymous to synonymous substitution rates (*ω*; dN/dS) in sawflies was calculated under a branch‐specific model using CodeML implemented in PAML v4.10.8 (Yang 2007). To assess positive selection across the entire phylogenetic gene tree, site model‐based analyses were conducted using M7 (*ω* ≤ 1) and M8 (*ω* > 1) models (Yang et al. 2000). Codon sites with a posterior probability exceeding 0.9 were considered as candidates for positive selection. Specifically, the branch‐ site model was used to detect positive selection occurring at individual codons in specific lineages. Particularly, the branch‐site model examined the likelihood ratio test (LRT), which compares the tested model (allows positive selection on each of the foreground lineages) with the null model that does not allow such positive selection. The branch‐site model was used to perform BEB analysis (model = 2, NSsites = 2) to identify positively selected sites in a given lineage (foreground), yielding posterior probabilities of 0.95 or 0.99. Likelihood ratio tests (LRT; 2Δℓ) between the models were calculated and the results were compared with chi‐square (*χ* ^2^) distribution thresholds calculated according to specific degrees of freedom. If the resulting 2Δℓ value was significant at the 1% (*p* < 0.01) or 0.1% (*p* < 0.001) level, the null model was rejected, and positive selection was considered statistically supported (Forni et al. 2024). The codons under pervasive and/or episodic positive selection for each gene were estimated using the codon‐based approaches FUBAR [the Bayesian MCMC‐based fast unbiased approximate Bayesian analysis (Murrell et al. 2013)] and MEME [mixed effects model of evolution (Murrell et al. 2012)] implemented in the HyPhy package on the DataMonkey server (http://www.datamonkey.org/dataupload.php). Statistical significance was assessed by posterior probability > 0.90 (FUBAR) and *p*‐value < 0.05 (MEME).

## 5 Conclusion

This study comprehensively identified and characterized the genes encoding the condensin I, condensin II, and cohesin complexes involved in the integrity and segregation of chromosomes across the suborder Symphyta (Hymenoptera). Between 10 and 12 genes and a total of 46 conserved motifs were predicted across six sawfly superfamilies. The absence of the *CAPG2* gene in certain species and the occurrence of gene copy number expansions in others suggests species‐specific variations in the evolutionary dynamics. The observed variation in the number of identified functional genes may be attributable to sporadic duplication events. The phylogenetic analyses are consistent with the hypothesis that *SMC* genes evolved through ancient duplication events and have been maintained under purifying selection pressure. In contrast, signals of positive selection detected in the *CAPG2*, *RAD21*, and *SA* genes suggest that specific regions of these genes have undergone adaptive evolution. Physicochemical properties and amino acid composition of these proteins are compatible with nucleoplasmic functions, playing key roles in DNA binding and protein–protein interactions. Given the limitations associated with genome assembly completeness and the use of transcriptome‐ derived based data, these findings should be considered preliminary and interpreted with caution. In conclusion, this study suggest that these complexes exhibit both conserved and lineage‐specific evolutionary characteristics in the symphytan species. Future studies focusing on the functional roles of positively selected regions and conducting comparative analyses with other insect orders could further elucidate the mechanisms of evolutionary adaptation underlying these essential chromosomal complexes.

## Author Contributions

**Ayşe Rümeysa Nalça:** formal analysis (equal), visualization (equal), writing – original draft (equal). **Ertan Mahir Korkmaz:** conceptualization (equal), data curation (equal), funding acquisition (equal), methodology (equal), project administration (equal), supervision (equal), validation (equal), writing – original draft (equal), writing – review and editing (equal).

## Funding

This work was supported by Sivas Cumhuriyet University Scientific Research Projects Coordination Unit.

## Conflicts of Interest

The authors declare no conflicts of interest.

## Supporting information


**Figure S1:** Nucleotide and amino acid composition of condensin and cohesin complex genes in symphytan species.

**Table S1:** Accession numbers and amino acid length of reference genes used in this study.
**Table S2:** Genome (SPAdes) and transcriptome (rnaSPAdes) assembly statistics of the symphytan species.
**Table S3:** Genome completeness evaluation of the symphytan species based on BUSCO analysis.
**Table S4:** Predicted gene numbers in symphytan species based on AUGUSTUS and TransDecoder annotations.
**Table S5:** Number of putatively functional condensin I, condensin II, and cohesin subunits among the symphytan species.
**Table S6:** Putative motif sequences and associated domains of condensin I–II and cohesin complex genes.
**Table S7:** Physicochemical properties of condensin I–II and cohesin complex genes in symphytan species.
**Table S8:** Comparison of nucleotide and amino acid sequence variability (%) among condensin I, condensin II, and cohesin complex genes (Figure 4).
**Table S9:** Gene‐ and species‐specific dN/dS (*ω*) ratios estimated for condensin I, condensin II, and cohesin complex genes among in symphytan species.
**Table S10:** Positively selected sites identified by MEME, FUBAR, and BEB analyses with corresponding LRT (2Δℓ) values from M7 vs. M8 models.
**Table S11:** Branch‐site model‐based likelihood ratio tests and positively selected sites in condensin and cohesin complex genes in symphytan species.

## Data Availability

The raw genomic sequencing data for *Xiphydria prolongata* have been deposited in the NCBI Sequence Read Archive (SRA) under the BioProject accession number PRJNA1345063. All nucleotide sequences generated in this study have been deposited in the NCBI GenBank database under the following accession numbers: *Xiphydria prolongata* (PX647654–PX647665), *Arge pagana* (PX653225–PX653236), *Neodiprion lecontei* (PX653237–PX653247), *Orussus abietinus* (PX653248–PX653259), *Syrista parreyssii* (PX653260–PX653270), *Tenthredo notha* (PX653271–PX653281), *Athalia japonica* (PX653282–PX653292), *Corynis zhengi* (PX653293–PX653303), *Cephalcia chuxiongica* (PX637991–PX638000), *Xyela alpigena* (PX638001–PX638012), and *Pergagrapta polita* (PX638013–PX638024). The genome/transcriptome assembly, and corresponding gene annotation files generated in this study are publicly available in the Zenodo repository: https://doi.org/10.5281/zenodo.20050222.
