# Supplementary figures and images for: Evolutionary Patterns of the Genes Involved in the Integrity and Segregation of Chromosomes in Sawflies (Hymenoptera: Symphyta)
Source: Ecol Evol. 2026 Jun 1;16(6):e73748. doi: 10.1002/ece3.73748 (PMC13239252; doi:10.1002/ece3.73748)

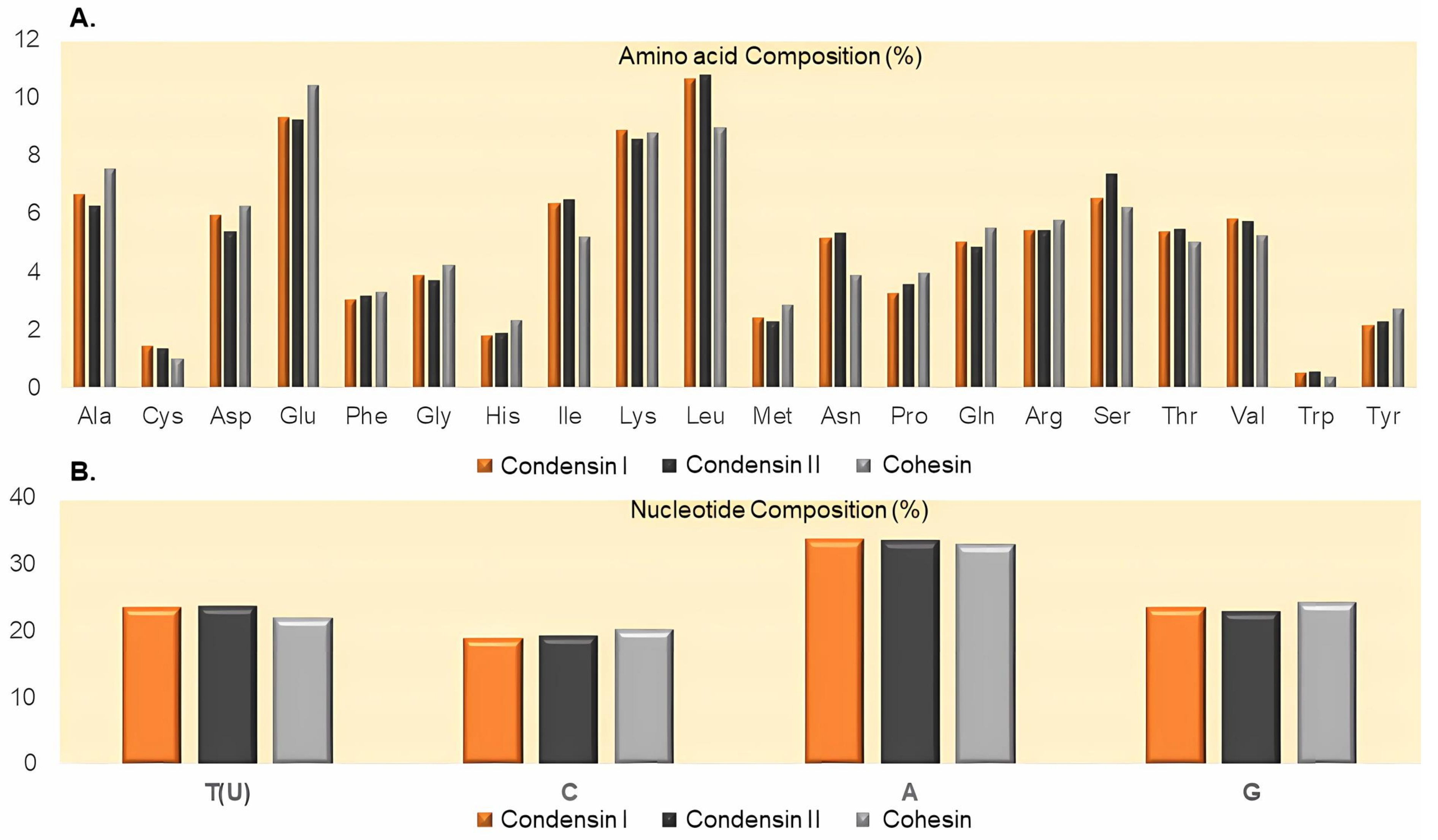

Supplement: Supplementary file 1 — Figure S1: Nucleotide and amino acid composition of condensin and cohesin complex genes in symphytan species. [file ECE3-16-e73748-s002.tif]
