# Supplementary material for: Evolutionary Patterns of the Genes Involved in the Integrity and Segregation of Chromosomes in Sawflies (Hymenoptera: Symphyta)
Source: Ecol Evol. 2026 Jun 1;16(6):e73748. doi: 10.1002/ece3.73748 (PMC13239252; doi:10.1002/ece3.73748)
Supplement: Supplementary file 2 — Table S1: Accession numbers and amino acid length of reference genes used in this study. Table S2: Genome (SPAdes) and transcriptome (rnaSPAdes) assembly statistics of the symphytan species. Table S3: Genome completeness evaluation of the symphytan species based on BUSCO analysis. Table S4: Predicted gene numbers in symphytan species based on AUGUSTUS and TransDecoder annotations. Table S5: Number of putatively functional condensin I, condensin II, and cohesin subunits among the symphytan species. Table S6: Putative motif sequences and associated domains of condensin I–II and cohesin complex genes. Table S7: Physicochemical properties of condensin I–II and cohesin complex genes in symphytan species. Table S8: Comparison of nucleotide and amino acid sequence variability (%) among condensin I, condensin II, and cohesin complex genes (Figure 4). Table S9: Gene‐ and species‐specific dN/dS (ω) ratios estimated for condensin I, condensin II, and cohesin complex genes among in symphytan species. Table S10: Positively selected sites identified by MEME, FUBAR, and BEB analyses with corresponding LRT (2Δℓ) values from M7 vs. M8 models. Table S11: Branch‐site model‐based likelihood ratio tests and positively selected sites in condensin and cohesin complex genes in symphytan species. [file ECE3-16-e73748-s001.pdf]

Supplementary Files

Supplementary Figure

Figure S1: Nucleotide and amino acid composition of condensin and cohesin complex genes in symphytan species

Supplementary Tables

Table S1: Accession numbers and amino acid length of reference genes used in this study

Table S2: Genome (SPAdes) and transcriptome (rnaSPAdes) assembly statistics of the symphytan species

Table S3: Genome completeness evaluation of the symphytan species based on BUSCO analysis

Table S4: Predicted gene numbers in symphytan species based on AUGUSTUS and TransDecoder annotations

Table S5: Number of putatively functional condensin I, condensin II, and cohesin subunits among the symphytan species

Table S6: Putative motif sequences and associated domains of condensin I-II and cohesin complex genes

Table S7: Physicochemical properties of condensin I-II and cohesin complex genes in symphytan species

Table S8: Comparison of nucleotide and amino acid sequence variability (%) among condensin I, condensin II, and cohesin complex genes (Fig. 4)

Table S9: Gene- and species-specific dN/dS (ω) ratios estimated for condensin I, condensin II, and cohesin complex genes among in symphytan species

Table S10: Positively selected sites identified by MEME, FUBAR, and BEB analyses with corresponding LRT ($2\Delta\ell$) values from M7 vs M8 models

Table S11: Branch-site model-based likelihood ratio tests and positively selected sites in condensin and cohesin complex genes in symphytan species

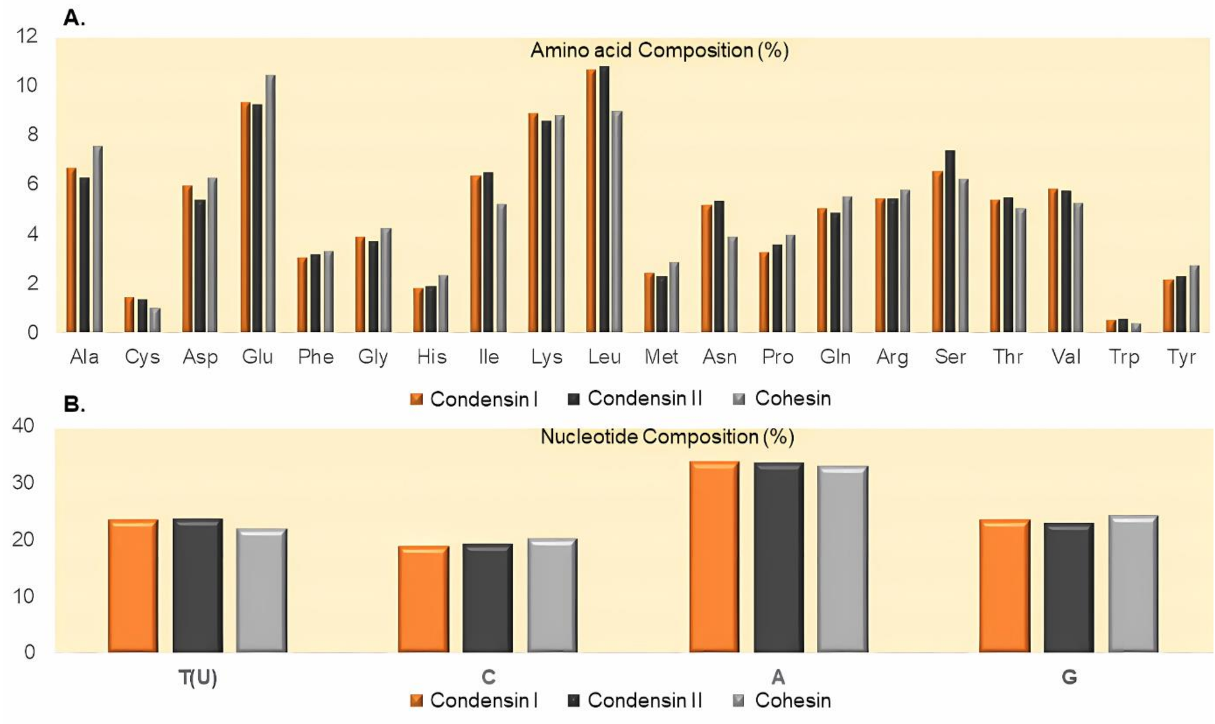

Figure S1: Nucleotide and amino acid composition of condensin and cohesin complex genes in symphytan species

Table S1: Accession numbers and amino acid length of reference genes used in this study

Gene name	Length (aa)	UniProt	Gene ID
**Condensin I**			
SMC2	1177	A0A7M7TFJ4	412832
SMC4	1362	A0A7M7R9L5	413418
CAPH	735	Q9VIP9	100576990
CAPD2	1380	Q9VAJ1	412839
CAPG	1351	A1Z987	36440
**Condensin II**			
CAPH2	1517	A0A7M7MNQ0	725563
CAPD3	1329	A0A7M7GA57	100578339
CAPG2	1156	A0A7M7GN29	100576754
**Cohesin**			
SMC1	1230	A0A8U0Z306	411589
SMC3	1202	A0A7M7TF53	410217
RAD21/Rec8	773	A0A7M7GQG0	411619
SA	1230	A0A7M7MRI7	727407

Table S2: Genome (SPAdes) and transcriptome (rnaSPAdes) assembly statistics of the symphytan species

Superfamily	Family	Species	Scaffold number (≥1000 bp)	N50 (kb)	L50 (kb)	Transcripts (≥1000 bp)	Longest transcript (bp)	N50 (kb)	GC (%)
Xyeloidea	Xyelidae	*X. alpigena*	–	–	–	10.552	21.576	1581	44.20
Tentredinoidea	Pergidae	*P. polita*	–	–	–	15.236	9715	1456	44.80
	Argidae	*A. pagana*	7661	84.275	630	–	–	–	43.39
	Athaliidae	*A. japonica*	9483	38.623	1189	–	–	–	40.54
	Cimbicidae	*C. zhengi*	9522	92.730	591	–	–	–	41.00
	Tenthredinidae	*T. notha*	407	2.089.156	30	–	–	–	37.18
	Diprionidae	*N. lecontei*	13.338	40.505	1559	–	–	–	39.59
Pamphilioidea	Pamphiliidae	*C. chuxiongica*	–	–	–	5548	26.078	531	36.90
Xiphydrioidea	Xiphydriidae	*X. prolongata*	22.903	37.901	1626	–	–	–	43.14
Cephoidea	Cephidae	*S. parreyssii*	1853	631.344	62	–	–	–	39.99
Orussoidea	Orussidae	*O. abietinus*	18.287	23.368	1515	–	–	–	45.42

Table S3: Genome completeness evaluation of the symphytan species based on BUSCO analysis

Superfamily	Family	Species	Complete BUSCO	Complete and single-copy BUSCO	Complete and duplicated BUSCO	Fragmented BUSCO	Missing BUSCO	Total BUSCO groups searched
Xyeloidea	Xyelidae	*X. alpigena*	3851	3358	493	530	1610	5991
Tentredinoidea	Pergidae	*P. polita*	3404	1914	1490	615	1972	5991
	Argidae	*A. pagana*	5366	5235	131	198	427	5991
	Athaliidae	*A. japonica*	5543	5519	24	215	233	5991
	Cimbicidae	*C. zhengi*	5682	5661	21	122	187	5991
	Tenthredinidae	*T. notha*	5624	5595	29	85	282	5991
	Diprionidae	*N. lecontei*	5459	5439	20	259	273	5991
Pamphilioidea	Pamphiliidae	*C. chuxiongica*	2670	2419	251	604	2717	5991
Xiphydrioidea	Xiphydriidae	*X. prolongata*	5359	5331	28	243	389	5991
Cephoidea	Cephidae	*S. parreyssii*	5806	5796	10	40	145	5991
Orussoidea	Orussidae	*O. abietinus*	5221	5217	4	290	480	5991

Table S4: Predicted gene numbers in symphytan species based on AUGUSTUS and TransDecoder annotations

Superfamily	Family	Species	Number of possible genes (AUGUSTUS v3.3.3)	Number of possible genes (TransDecoder v5.5.0)
Xyeloidea	Xyelidae	*X. alpigena*	–	21.576
Tentredinoidea	Pergidae	*P. polita*	–	9.715
	Argidae	*A. pagana*	16.554	–
	Athaliidae	*A. japonica*	17.747	–
	Cimbicidae	*C. zhengi*	46.891	–
	Tenthredinidae	*T. notha*	30.158	–
	Diprionidae	*N. lecontei*	20.276	–
Pamphilioidea	Pamphiliidae	*C. chuxiongica*	–	26.078
Xiphydrioidea	Xiphydriidae	*X. prolongata*	30.982	–
Cephoidea	Cephidae	*S. parreyssii*	21.059	–
Orussoidea	Orussidae	*O. abietinus*	15.818	–

Table S5: Number of putatively functional condensin I, condensin II, and cohesin subunits among the symphytan species

Superfamily	Family	Species	Condensin I					Condensin II			Cohesin				Total
			SMC2	***SMC4***	***CAPH***	***CAPD2***	***CAPG***	***CAPH2***	***CAPD3***	***CAPG2***	***SMC1***	***SMC3***	***RAD21***	***SA***	
Xyeloidea	Xyelidae	*X. alpigena*	1	1	1	1	1	1	1	1	1	1	1	1	12
Tentredinoidea	Pergidae	*P. polita*	1	1	1	1	1	1	1	1	1	1	1	1	12
	Argidae	*A. pagana*	1	1	2	1	1	1		1	1	1	1	1	13
	Athaliidae	*A. japonica*	1	1	1	1	1	1	2	-	1	1	1	1	12
	Cimbicidae	*C. zhengi*	1	1	1	1	2	1	1	-	1	1	1	1	12
	Tenthredinidae	*T. notha*	1	1	1	1	1	1	1	-	2	1	1	1	12
	Diprionidae	*N. lecontei*	1	1	1	2	1	1	1	-	1	2	1	2	14
Pamphilioidea	Pamphiliidae	*C. chuxiongica*	1	1	1	1	1	-	-	1	1	1	1	1	10
Xiphydrioidea	Xiphydriidae	*X. prolongata*	1	1	2	1	1	1	1	1	1	1	1	1	13
Cephoidea	Cephidae	*S. parreyssii*	2	2	2	2	1	1	1	-	2	1	2	2	14
Orussoidea	Orussidae	*O. abietinus*	1	1	1	1	2	1	1	1	2	1	1	1	14
		Total	**12**	**12**	**14**	**13**	**13**	**10**	**11**	**6**	**14**	**12**	**12**	**13**	**142**
		Single-copy	11	11	11	11	11	10	10	6	11	11	11	11	125

Table S6: Putative motif sequences and associated domains of condensin I-II and cohesin complex genes

Gene	Motif No	Width (aa)	Consensus Sequence	Domain (Pfam/NCBI-CDD)
SMC1	Motif-I	50	PYLDGINYNCVAPGKRFQPMSNLSGGEKTVAALALLFAIHSFQPAPFFVL	SMC_N
	Motif-II	50	ALDNTNIGKVASYIRDKTSSLQTIVISLKEEFYSHADALIGICPDVGECL	SMC_N
	Motif-III	50	RLFPGVYDRMYNMCEPIHKRYNVAITKVLGKYMEAIVVDTEKTARQCIQY	SMC_hinge
	Motif-IV	50	FTAVVGPNGSGKSNFMDAISFVMGEKTSSLRVKRFSELIHGASIGMPVAR	SMC_N
SMC2	Motif-I	50	AMTWIFGQIFICKDIEIAKKIAFHDNIMKKCVTLEGDVVDPAGILSGGAP	SMC_hinge
	Motif-II	50	HFESKYPQIRFEYQKPDSNFNHNSVKGVVCKLITVKDKNAAYALDIAAGG	SMC_hinge
	Motif-III	50	TLLKAWEQVNKDFGSIFSSLLPGADAKLQPCENQTVTEGLEIKIAFSGIW	SMC_N
	Motif-IV	50	QHGQLQKRVTIIPLNKVGGKSMDNQLIHLAQKIGGIENVRPALSLIDFPE	SMC_hinge
	Motif-V	50	SLGELSGGQRSLVALSLILAMLLFKPAPLYILDEVDAALDLSHTENIGIM	SMC_N
SMC3	Motif-I	50	TFMPLNRLHVKDIDYPETSDAIPMISQLNYDQKYDKALRYIFGKTLICRN	SMC_hinge
	Motif-II	50	TRKDMAHEVSSYYGPVIENFSCDKSVYMAVEVTAGNRLFHHIVETDKFGT	SMC_hinge
	Motif-III	50	IFAIQKCDPAPFYLFDEIDQALDAQHRKAVADMIHELSSDAQFITTTFRP	SMC_N
	Motif-IV	50	KLVKRKEELDRGDEKIKELMSVLEQRKCEAIQFTFKQVSKYFSEVFKKLV	Smc
SMC4	Motif-I	50	DPFSEGIAFSVRPPKKSWKNICNLSGGEKTLSSLALVFALHHYKPTPLYF	SMC_N
	Motif-II	50	AQFIIISLRSNMFELADYLVGIYKTYNCTKSVTVDLKKYYEKNGIAPPTQ	SMC_N
	Motif-III	50	QNFKSYGGTQVIGPFHQCFSAIVGPNGSGKSNVIDSMLFVFGYRASKIRS	SMC_N
	Motif-IV	50	AVSTACGPLDNIVVDTVTTAQMCITFLRQNDIGRATFIPLEKQQRFLSRC	SMC_hinge
	Motif-V	50	RNRIRDIYETTRRRKIQEFLAGFTIITDKLKEMYQMITLGGDAELELVDS	SMC_N
	Motif-VI	50	VQFKEIAKILRFYGVDLDHNRFLILQGEVEQIAMMKPKAQHENDTGMLEF	SMC_N
	Motif-VII	50	PENVPRLFDLIRVEDERVLPAFYYGLQDTLVADNLDQATRIAYGHKRFRV	SMC_hinge
	Motif-VIII	50	HNSSEHQNINSCTVSVHFQQIIDKLEMEYDIVPNSEIVISRTAFKDNSSY	SMC_N
CAPH	Motif-I	50	TIKNCLELYNGNKVSKDNAWNLMLIDSLANLLDHHHKRMSNFKMAGSSLE	Cnd2

	Motif-II	50	TIPSHPKKKGEHYSKGFASFQQVYQKLPDLLTTKMSDSLSPSVAFYAVLH	Cnd2
	Motif-III	41	GTEFEGAPSQVAKVIVPFAKRAKVIDMKNLKKSCNSLIQKQ	Cnd2
	Motif-IV	50	QTEFIEDLRPVDGNSKLEYSYRPMDQISQFWAGPSHWKFKR	Cnd2
	Motif-V	50	DYGNVTENFFQQLDTTIRQRKANFQKKWDPRKLILPTKFELDPDLFFKYE	Cnd2
	Motif-VI	29	ASSKVYGLRVDSIYLDAMRISAGLSARTL	Cnd2
	Motif-VII	41	GVSRMLTPFNDDEAERREARRRTLLQQHHRSSTLESIEDNE	Cnd2
	Motif-VIII	21	LANDLKLRLIPQEDLEDFQIR	Cnd2
CAPD2	Motif-I	50	FCESNMSFLMNILNLTKNIRIKCNTVVGLSDLTFRFPNIIEPWTGHFYAQ	Cnd1
	Motif-II	50	HESNTELRLTAVKMLSHLILNEMIRVKGQIADMALCIVDGNEEIRNITKQ	Cnd1
	Motif-III	50	CLRFPVTRVERQWRDIAYCLGLLTYNERAVKKLMDNMQHYRDKVQVDEVY	Cnd1
	Motif-IV	50	KSNILYNVLPDIISRLGDINLNLDEDKYRIIMSYILGLIQKDRQIETLVE	Cnd1
CAPG	Motif-I	50	VGAYMYHLTSDPSPNVRQCIITCMGRNYITIPHILERLWDVDEKVRRHTY	Cnd3
	Motif-II	50	NMCNYPVRSYKVAQRLTLLEQGLNDTSATVRKTVINFMLKTWIESYQQNY	HEAT
	Motif-III	50	YYMTASPHVHKLTPSLCQLYKDFICRYLPCAEVDIFEWSIKCGTTFSIFY	Cnd3
	Motif-IV	50	RRLNKHSLLQPCFLPTVWTCNYDSPLHGVQPEHLTKFFLEMTVQEMSTPQ	Cnd3
CAPH2	Motif-I	50	HKMKEIFEESNVTTELDQTVAKWHASLQPKLSEVETRPTFRIHDYSSRII	CNDH2_C
	Motif-II	29	QKTVNFDTIVQDKPAYEVARYFLASLQLI	CNDH2_C
CAPD3	Motif-I	50	RFTALVEPYLPDMCVSMKDSNPQVREAIMVIFIQLLLEDYIKVKGPFFFH	Cnd1
	Motif-II	50	HEVLSVISLRCRDPVLIVRRFAVQVLSQLLQEFPNNSQLLNEWVRTVMPQ	HEAT
CAPG2	Motif-I	50	AGRGKLGKNLLSLLTAIHNNKQQVARSMIHNQCKPLLWKHLKAPGSFIRC	Condensin2nSMC
RAD21	Motif-I	50	NKRASHMYHVIKSKLDTKDRLTLSEMAYKNNRKQVAQKFYTLLVLKKFQV	Rad21_Rec8
	Motif-II	50	MALRTSGHLLLGVVRIYSRKAKYLLADCNEAFVKIKMAFRPGMVDLPEEH	Rad21_Rec8_N
SA	Motif-I	50	RVVLNFLDRRLQAGMPSSRGEDWQPLLLYRNSLLHGETDQVPVTSKRAYT	Scc3/SA
	Motif-II	50	YDVAVQAVKLVISILKHHREILTDKDCEHVYELVYSSHRAVAQAAGEFLN	Scc3/SA

Table S7: Physicochemical properties of condensin I-II and cohesin complex genes in symphytan species

Superfamily	Family	Species	Gene	Length (aa)	Molecular Weight (kDa)	Instability Index	Aliphatic Index	pI	Hydropathicity (GRAVY)
Xyeloidea	Xyelidae	*X. alpigena*	*SMC2*	985	112936.81	48.30	86.26	7.34	-0.768
			SMC4	656	74363.84	48.59	84.84	6.53	-0.704
			CAPH	213	23592.29	25.64	71.32	4.69	-0.620
			CAPD2	399	45327.09	53.52	88.67	7.70	-0.512
			CAPG	314	36325.52	41.89	102.83	8.39	-0.085
			CAPH2	555	62968.86	56.49	69.33	5.12	-0.945
			CAPD3	532	60476.71	59.92	86.25	8.75	-0.559
			CAPG2	160	18053.76	47.96	94.56	9.17	-0.322
			SMC1	1227	141336.08	41.84	76.15	6.16	-0.873
			SMC3	1029	119121.80	48.07	75.01	7.177	-0.921
			RAD21	761	83514.26	61.14	71.92	4.72	-0.532
			SA	738	84306.96	39.29	89.84	5.32	-0.406
Tentredinoidea	Pergidae	*P. polita*	*SMC2*	567	64959.41	38.74	85.51	8.78	-0.749
			SMC4	1357	154425.90	43.98	85.37	6.44	-0.640
			CAPH	667	75655.20	45.33	85.63	5.40	-0.557
			CAPD2	483	54618.89	62.49	78.13	8.71	-0.683
			CAPG	287	32937.96	42.79	104.25	4.72	-0.207
			CAPH2	566	14459.03	53.67	76.85	4.99	-0.791
			CAPD3	528	58744.82	53.31	92.14	6.70	-0.311
			CAPG2	700	79815.05	43.75	111.33	5.89	0.124
			SMC1	1232	141667.54	45.38	45.38	6.25	-0.831
			SMC3	642	73483.40	40.02	75.68	6.05	-0.710

			RAD21	446	49595.07	62.72	75.87	4.97	-0.584
			SA	381	43225.06	66.39	83.39	6.66	-0.578
			SMC2	1541	176843.43	40.24	87.90	6.47	-0.568
			SMC4	1375	155870.71	46.30	84.56	6.84	-0.671
			CAPH	661	75541.67	47.68	80.08	5.48	-0.736
			CAPD2	1455	163623.65	43.36	95.99	6.31	-0.360
			CAPG	2331	269205.16	47.41	84.43	5.96	-0.525
	Argidae	*A. pagana*	*CAPH2*	612	69632.05	63.24	71.70	5.34	-0.789
			CAPD3	1313	148026.87	44.25	100.56	6.04	-0.160
			CAPG2	1154	131687.10	35.99	111.64	6.82	0.038
			SMC1	1186	136073.02	46.58	77.01	6.06	-0.826
			SMC3	1197	138426.16	42.79	75.88	8.30	-0.862
			RAD21	786	86482.45	58.40	77.07	4.73	-0.457
			SA	1166	134318.59	40.95	86.45	5.78	-0.444
			SMC2	1179	133771.17	38.57	86.88	8.68	-0.634
			SMC4	1357	154286.24	46.75	83.15	6.47	-0.655
			CAPH	688	77150.42	42.69	75.82	5.33	-0.660
			CAPD2	1703	191723.00	47.77	92.99	7.22	-0.396
			CAPG	922	105563.06	44.47	105.20	5.32	-0.131
	Athaliidae	*A. japonica*	*CAPH2*	607	68960.30	54.88	76.93	6.14	-0.723
			CAPD3	1307	149029.14	47.53	104.13	5.87	-0.183
			CAPG2	-	-	-	-	-	-
			SMC1	1260	144825.17	45.25	77.72	6.50	-0.852
			SMC3	1147	132191.12	44.37	77.75	8.34	-0.851
			RAD21	783	86100.00	60.05	74.04	4.73	-0.493
			SA	2018	230160.10	47.01	81.34	6.16	-0.502
	Cimbicidae	*C. zhengi*	*SMC2*	2943	329025.65	47.83	76.73	7.37	-0.511

			SMC4	1491	169852.51	44.78	83.44	7.04	-0.665
			CAPH	700	19258.24	47.50	90.75	9.75	-0.339
			CAPD2	1337	152813.90	47.57	102.33	6.29	-0.263
			CAPG	916	105276.95	46.68	108.89	5.61	-0.128
			CAPH2	611	69593.74	44.02	76.25	6.23	-0.673
			CAPD3	1340	152677.14	46.71	102.17	5.98	-0.157
			CAPG2	-	-	-	-	-	-
			SMC1	1300	149075.36	43.84	76.51	6.76	-0.800
			SMC3	1203	138790.70	44.05	77.85	8.11	-0.821
			Rad21	794	87302.38	58.91	75.69	4.77	-0.491
			SA	1249	143095.55	41.12	83.01	5.77	-0.528
	Tenthredinidae	*T. notha*	*SMC2*	2505	277692.17	46.82	76.94	7.87	-0.480
			SMC4	2207	246883.13	50.63	80.76	6.50	-0.535
			CAPH	698	78517.68	38.76	77.86	4.95	-0.713
			CAPD2	1246	140828.31	43.72	100.55	5.72	-0.179
			CAPG	927	106379.92	48.92	107.59	5.08	-0.139
			CAPH2	644	72502.89	56.21	74.65	5.00	-0.684
			CAPD3	1371	155018.36	46.42	101.91	7.00	-0.214
			CAPG2	-	-	-	-	-	-
			SMC1	1263	80430.58	43.81	74.81	9.04	-0.910
			SMC3	1067	139249.20	43.61	83.46	7.04	-0.727
			RAD21	783	85857.67	60.41	75.40	4.72	-0.487
			SA	1278	146760.98	44.21	80.64	5.95	-0.569
	Diprionidae	*N. lecontei*	*SMC2*	2448	270203.02	48.23	73.25	6.54	-0.501
			SMC4	1915	212876.60	51.53	80.10	6.54	-0.579
			CAPH	687	75929.33	40.48	71.38	5.15	-0.674
			CAPD2	1123	127163.95	45.34	98.14	6.36	-0.260

			CAPG	991	113334.34	47.10	102.32	5.70	-0.128
			CAPH2	647	73550.12	51.61	77.49	5.16	-0.729
			CAPD3	1363	154453.01	45.52	99.91	6.00	-0.190
			CAPG2	-	-	-	-	-	-
			SMC1	1275	146223.90	43.98	77.82	6.68	-0.822
			SMC3	1218	140623.00	43.42	81.13	7.96	-0.761
			RAD21	786	86417.29	58.04	74.08	4.72	-0.505
			SA	1271	145468.69	47.75	82.95	5.61	-0.494
Pamphilioidea	Pamphiliidae	*C. chuxiongica*	*SMC2*	857	97747.37	38.06	86.72	7.94	-0.709
			SMC4	415	46940.55	50.23	84.13	7.74	-0.629
			CAPH	247	28202.43	41.89	67.13	4.87	-0.883
			CAPD2	969	108141.84	40.92	88.11	7.27	-0.451
			CAPG	135	15752.31	35.15	91.78	9.51	-0.346
			CAPH2	-	-	-	-	-	-
			CAPD3	-	-	-	-	-	-
			CAPG2	396	45216.06	44.34	114.65	6.79	0.155
			SMC1	514	58938.13	51.22	75.36	5.14	-0.849
			SMC3	675	77265.79	38.31	38.31	6.62	-0.672
			RAD21	267	29441.17	67.94	71.13	4.49	-0.595
			SA	1294	147871.03	46.65	78.91	5.83	-0.552
Xiphydrioidea	Xiphydriidae	X. prolongata	*SMC2*	1801	201241.04	43.80	84.32	6.51	-0.577
			SMC4	878	99035.03	42.57	87.29	6.35	-0.579
			CAPH	685	73862.91	52.96	75.04	5.15	-0.637
			CAPD2	1391	156499.02	47.77	98.24	5.91	-0.313
			CAPG	935	106825.68	44.54	96.03	5.05	-0.247
			CAPH2	1504	168234.66	48.91	81.58	5.68	-0.341
			CAPD3	1190	135564.72	42.36	104.79	5.80	-0.119

			CAPG2	977	111909.72	42.11	110.36	5.8	-0.022
			SMC1	1195	137395.86	45.78	75.84	6.34	-0.853
			SMC3	1203	138910.58	43.75	78.91	7.98	-0.829
			RAD21	753	82180.24	58.57	70.99	4.82	-0.568
			SA	1054	120970.17	42.81	84.30	5.62	-0.485
Cephoidea	Cephidae	*S. parreyssii*	*SMC2*	1202	137.79	45.25	88.12	8.44	-0.688
			SMC4	1476	167.43	50.28	84.02	6.31	-0.697
			CAPH	690	78.16	43.14	72.77	5.25	-0.701
			CAPD2	751	165.40	45.90	94.72	6.10	-0.412
			CAPG	918	106.09	48.17	98.04	5.23	-0.245
			CAPH2	751	85.79	60.22	69.40	5.22	-0.842
			CAPD3	1344	152.38	43.33	103.51	6.34	-0.174
			CAPG2	-	-	-	-	-	-
			SMC1	1533	176.36	47.64	78.77	6.54	-0.744
			SMC3	1421	164.10	42.09	76.05	6.30	-0.827
			RAD21	786	85.73	60.36	75.02	4.78	-0.495
			SA	1298	147.43	46.34	80.24	5.75	-0.528
Orussoidea	Orussidae	*O. abietinus*	*SMC2*	1168	133.59	39.59	90.02	8.26	-0.657
			SMC4	1340	152759.47	46.11	85.20	6.62	-0.630
			CAPH	690	77100.90	48.63	75.96	4.97	-0.799
			CAPD2	1178	131340.27	43.23	101.92	7.93	-0.277
			CAPG	918	17855.14	25.98	105.68	9.48	-0.054
			CAPH2	751	91839.96	53.28	66.63	4.75	-0.796
			CAPD3	1344	153606.42	45.38	97.71	6.40	-0.214
			CAPG2	1086	124060.17	40.71	111.91	8.85	0.024
			SMC1	1230	141377.55	43.50	79.55	6.45	-0.820
			SMC3	1211	139724.84	43.79	79.83	8.37	-0.801

			RAD21	786	85343.30	63.67	73.57	4.74	-0.486
			SA	1298	146091.80	44.29	79.39	5.62	-0.581

Table S8: Comparison of nucleotide and amino acid sequence variability (%) among condensin I, condensin II, and cohesin complex genes (Fig. 4)

**Gene**	**Nucleotid variable**		**Amino acid variable**	
	**Variable**	**%**	**Variable**	**%**
All genes	36948/39675	0.931	9284/13225	0.702
**Condensin I**				
SMC2	2386/3522	0.677	785/1174	0.668
SMC4	2824/4377	0.645	872/1459	0.597
CAPD2	3311/4293	0.771	1163/1431	0.812
CAPG	2023/2745	0.736	704/915	0.769
CAPH	1547/1902	0.813	532/634	0.839
**Condensin II**				
CAPD3	3038/4038	0.752	1032/1346	0.766
CAPG2	2294/3366	0.681	860/1122	0.766
CAPH2	1711/2013	0.849	590/671	0.879
**Cohesin**				
SMC1	1698/3645	0.465	337/1215	0.277
SMC3	1632/3612	0.451	273/1204	0.226
RAD21	1190/2343	0.507	294/781	0.376
SA	1917/3819	0.501	429/123	0.336

Table S9: Gene- and species-specific dN/dS (ω) ratios estimated for condensin I, condensin II, and cohesin complex genes among in symphytan species

ω=dN/dS														
**Superfamily**	**Family**	**Species**	**Condensin I**					**Condensin II**			**Cohesin**			
			SMC2	***SMC4***	***CAPH***	***CAPG***	***CAPD2***	***CAPH2***	***CAPG2***	***CAPD3***	***SMC1***	***SMC3***	***RAD21***	***SA***
Xyeloidea	Xyelidae	*X. alpigena*	0.0538	0.0659	0.0865	0.0997	0.1226	0.1517	0.1488	0.1028	0.0201	0.0111	0.0488	0.0297
Tentredinoidea	Pergidae	*P. polita*	0.0554	0.0660	0.0839	0.0994	0.1226	0.1524	0.1521	0.1081	0.0201	0.0114	0.0477	0.0285
	Argidae	*A. pagana*	0.0544	0.0671	0.0861	0.1000	0.1253	0.1571	0.1608	0.1087	0.0198	0.0124	0.0478	0.0288
	Athaliidae	*A. japonica*	0.0560	0.0668	0.0862	0.0979	0.1146	0.1427	-	0.1031	0.0186	0.0126	0.0497	0.0295
	Cimbicidae	*C. zhengi*	0.0526	0.0675	0.0771	0.0870	0.1138	0.1451	-	0.0941	0.0181	0.0133	0.0481	0.0284
	Tenthredinidae	*T. notha*	0.0565	0.0676	0.0952	0.0971	0.1140	0.1537	-	0.1086	0.0192	0.0134	0.0499	0.0307
	Diprionidae	*N. lecontei*	0.0599	0.0657	0.0849	0.0949	0.1157	0.1390	-	0.1086	0.0180	0.0126	0.0494	0.0279
Pamphilioidea	Pamphiliidae	*C. chuxiongica*	0.0529	0.0668	0.0871	0.1029	0.1215	-	0.1515	-	0.0187	0.0119	0.0465	0.0298
Xiphydrioidea	Xiphydriidae	*X. prolongata*	0.0599	0.0697	0.0867	0.1005	0.1251	0.1638	0.1376	0.1049	0.0208	0.0123	0.0509	0.0290
Cephoidea	Cephidae	S. parreyssii	0.0592	0.0738	0.1067	0.1050	0.1289	0.1656	-	0.1101	0.0205	0.0111	0.0494	0.0321
Orussoidea	Orussidae	*O. abietinus*	0.0552	0.0678	0.0830	0.0997	0.1211	0.1573	0.1595	0.1129	0.0192	0.0126	0.0510	0.0302
		Avarage	**0.0560**	**0.0704**	**0.0876**	**0.0985**	**0.1187**	**0.1528**	**0.1517**	**0.1062**	**0.0194**	**0.0122**	**0.0489**	**0.0295**

Table S10: Positively selected sites identified by MEME, FUBAR, and BEB analyses with corresponding LRT (2Δℓ) values from M7 vs M8 models

**Gene**	**MEME (P ≤ 0.05)**	**FUBAR (P ≥ 0.90)**	**BEB (BEB > 0.95) M7/M8 model (site model)**	**2Δl (LRT)**
**Condensin I**				
SMC2	347, 353, 841, 1165	-	-	11.588
SMC4	399, 695, 1373, 1438	-	-	3.919
CAPD2	11, 215, 284, 408, 477, 517, 526, 531, 618, 662, 945, 959, 1136, 1286, 1322, 1362	-	-	17.431
CAPH	52, 110, 174, 493, 670	-	-	0.241
CAPG	51, 446, 540, 544, 685, 808	142	-	25.780
**Condensin II**				
CAPD3	72, 177, 185, 231, 294, 457, 714, 1022, 1194, 1299	-	-	15.211
CAPH2	214, 228, 607	202	-	4.374
CAPG2	25, 74, 101, 383, **412**, 518, 855, 984, 1024	**412**	-	3.086
**Cohesin**				
SMC1	520, 862, 982	-	-	8.689
SMC3	3, 354, 908	-	-	0
RAD21	**344**, 688	-	-	2.92
SA	772, 870, 1056, 1086, 1164, 1250, **1266**	1182	-	7.00

Table S11: Branch-site model-based likelihood ratio tests and positively selected sites in condensin and cohesin complex genes in symphytan species

Superfamily	Family	Species	Gene	lnL (null model)	lnL (alternative model)	2Δl (LRT)	Significance	ω₂a	Positively selected sites (BEB) (BEB > 0.95)
Xyeloidea	Xyelidae	*X. alpigena*	*SMC1*	-23938.8	-23936.7	4.22	p < 0.05	2.00	27S*, 855R*, 877S*, 913Q*, 989P*
			SMC2	-31145.8	-31137.0	17.43	p < 0.01	41.49	527C*, 550Y*, 681L*
			SMC3	-22224.9	-22224.9	0	ns	1.00	265Q**, 600I*, 822L*, 1092H*
			SMC4	-33136.9	-33133.6	6.686	p < 0.05	1.00	-
			CAPD2	-38833.1	-38833.1	0	ns	107.93	-
			CAPD3	-35460.9	-35460.9	0	ns	1.00	-
			CAPG	-22511.8	-22513.8	0	ns	1.00	-
			CAPG2	-18266.6	-18263.9	5.3898	p < 0.05	20.84	-
			CAPH	-21346.1	-21342.6	6.911	p < 0.01	14.73	-
			CAPH2	-18648.5	-18641.6	13.74	p < 0.001	4.21	-
			RAD21	-16263.1	-16263.1	0.103	ns	1.97	-
			SA	-25080.4	-25080.4	0	ns	1.00	-
Tentredinoidea	Pergidae	*P. polita*	*SMC1*	-23991.7	-23988.7	6.13	p < 0.05	62.69	970N*
			SMC2	-31145.8	-31143.2	5.15*	p < 0.05	1.00	880E*
			SMC3	-22262.0	-22262.9	0.033	ns	1.19	714S*
			SMC4	-33107.8	-33107.7	2.183	ns	1.18	758D*, 1153D*, 1396P*
			CAPD2	-38829.4	-38828.8	1.15	ns	1.00	-

			CAPD3	-35462.3	-35461.1	2.54	ns	3.51	-
			CAPG	-22509.7	-22513.8	0	ns	1.00	-
			CAPG2	-18264.5	-18263.4	2.30	ns	26.54	-
			CAPH	-21343.0	-21341.8	2.390	ns	4.33	-
			CAPH2	-18658.4	-18657.4	2.02	ns	2.26	-
			RAD21	-16261.5	-16260.9	0.267	ns	87.55	711A*
			SA	-25049.6	-25076.0	52.81	p < 0.0001	9.96	896N*, 908Q*
	Argidae	*A. pagana*	*SMC1*	-23991.1	-23991.2	0	ns	28.19	-
			SMC2	-31142.4	-31142.4	0	ns	1.00	-
			SMC3	-22275.6	-22275.4	0.419	ns	25.16	-
			SMC4	-33129.7	-33129.7	0	ns	1.00	-
			CAPD2	-38816.9	-38803.7	26.5034	p < 0.001	12.24	1431D*
			CAPD3	-35458.7	-35454.5	8.454	p < 0.005	77.85	1222T*
			CAPG	-22507.1	-22507.1	4.065	p < 0.05	58.47	-
			CAPG2	-18266.0	-18266.0	0	ns	1.00	-
			CAPH	-21338.1	-21334.8	6.648	p < 0.01	50.04	-
			CAPH2	-18662.1	-18659.8	4.295	p < 0.05	363.41	-
			RAD21	-16260.0	-16258.7	2.71	ns	68.15	**344P***
			SA	-25077.9	-25077.3	11.607	ns	11.58	-
	Athaliidae	*A. japonica*	*SMC1*	-23974.5	-23969.7	9.707	p < 0.01	48.54	904A*, 957S*
			SMC2	-31139.7	-31137.9	3.585	ns	8.22	120S*
			SMC3	-22280.0	-22280.0	0	ns	1.00	-
			SMC4	-33131.7	-33130.7	1.910	ns	2.88	-
			CAPD2	-38808.3	-38804.2	8.07	p < 0.01	101.37	1262Q*, 1273C*, 1294D*, 1297T*
			CAPD3	-35451.5	-35450.7	1.64	ns	2.35	-
			CAPG	-22505.6	-22505.3	0.63	ns	1.46	355E*

			CAPH	-21336.9	-21333.9	5.836	$p < 0.05$	170.84	-
			CAPH2	-18662.6	-18662.6	0	ns	1.00	-
			RAD21	-16262.9	-16262.5	0.63	ns	3.17	-
			SA	-25078.3	-25069.1	18.47	$p < 0.005$	1.18	629I*, 699H**
	Cimbicidae	*C. zhengi*	*SMC1*	-23991.7	-23982.6	0	ns	1.00	-
			SMC2	-31129.4	-31129.4	0	ns	1.00	179Q*, 469D*, 774N*, 971Q*
			SMC3	-22280.0	-22280.0	0	ns	1.00	-
			SMC4	-33136.0	-33136.0	0	ns	1.00	-
			CAPD2	-38830.8	-38830.8	0	ns	1.00	-
			CAPD3	-35453.7	-35453.7	0	ns	1.00	-
			CAPG	-22488.2	-22505.3	0	ns	1.00	355E*
			CAPH	-21335.4	-21334.4	2.037	ns	2.61	520C*
			CAPH2	-18654.2	-18654.2	0	ns	1.00	-
			RAD21	-16262.8	-16262.5	0.55	ns	13.5	543S*
			SA	-25075.1	-25074.0	2.29	ns	3.87	-
	Tenthredinidae	*T. notha*	*SMC1*	-23990.6	-23991.7	0	ns	26.80	-
			SMC2	-31137.4	-31135.4	3.961	$p < 0.05$	45.62	78T*, 395N*, 1167T*
			SMC3	-22277.4	-22275.8	3.250	ns	30.94	748S*
			SMC4	-33136.0	-33136.9	0	ns	1.00	-
			CAPD2	-38822.8	-38822.3	1.09	ns	1.24	-
			CAPD3	-35463.1	-35462.0	2.16	ns	3.23	-
			CAPG	-22510.6	-22510.0	1.02	ns	2.43	-
			CAPH	-21336.0	-21334.3	5.258	$p < 0.05$	29.45	-
			CAPH2	-18666.5	-18666.6	0	ns	1.00	-
			RAD21	-16264.1	-16264.1	0	ns	1.00	-
			SA	-25081.4	-25079.3	4.23	$p < 0.05$	89.59	790P*

	Diprionidae	*N. lecontei*	*SMC1*	-3972.3	-23972.0	0.637	ns	1.49	-
			SMC2	-31144.9	-31144.6	0.601	ns	2.17	-
			SMC3	-22271.1	-22268.2	5.778	$p < 0.02$	20.84	-
			SMC4	-33129.8	-33127.1	55.934	$p < 0.05$	60.08	495D*, 657A*
			CAPD2	-38826.9	-38823.1	74.924	$p < 0.01$	10.66	501A*, 962N*
			CAPD3	-35460.2	-35459.0	2.43	ns	3.05	-
			CAPG	-22503.3	-22499.9	6.71	$p < 0.01$	43.90	16F*, 20Q, 592L*, 880K**
			CAPH	-21345.5	-21345.5	0	ns	1.00	-
			CAPH2	-18666.5	-18666.5	0	ns	1.00	-
			RAD21	-16264.1	-16264.1	0	ns	3.80	-
			SA	-25078.3	-25078.3	0	ns	1.00	-
Pamphilioidea	Pamphiliidae	*C. chuxiongica*	*SMC1*	-23991.7	-23991.1	1.26	ns	1.16	-
			SMC2	-31145.8	-31127.9	35.60	$p < 0.001$	2.65	375T*, 422T*, 590T*, 740K*, 810E*, 820Q**, 887K*, 1034W*
			SMC3	-22271.8	-22271.8	0	ns	1.00	-
			SMC4	-33133.4	-33132.4	2.089	ns	65.77	1123P*
			CAPD2	-38833.0	-38831.9	4.07	$p < 0.05$	72.67	-
			CAPG	-22513.8	-22513.8	0	ns	1.00	-
			CAPG2	-18265.2	-18265.2	0	ns	1.00	-
			CAPH	-21338.5	-21342.6	0	ns	4.68	-
			RAD21	-16247.2	-16247.2	0	ns	1.00	443G**, 612A*, 624E*
			SA	-25077.6	-25080.0	0	ns	1.00	-
Xiphydrioidea	Xiphydriidae	*X. prolongata*	*SMC1*	-23987.7	-23986.7	1.99	ns	28.72	215E*
			SMC2	-31126.6	-31124.1	4.858	$p < 0.05$	30.62	4K*, 5S*
			SMC3	-22278.0	-22277.9	0.131	ns	1.56	-
			SMC4	-33120.1	-33127.3	0	ns	51.69	411D*, 618A*

			CAPD2	-38821.3	-38817.8	7.010	p < 0.01	3.31	-
			CAPD3	-35457.1	-35457.1	0	ns	1.00	-
			CAPG	-22503.6	-22503.0	1.150	ns	1.75	-
			CAPG2	-18260.7	-18260.7	0	ns	1.03	-
			CAPH	-21341.6	-21340.9	1.425	ns	36.26	-
			CAPH2	-18665.2	-18663.9	2.601	p < 0.05	65.33	-
			RAD21	-16226.3	-16221.5	9.44	p < 0.005	49.70	241G*, 284E*, 298S*, 305S*
			SA	-25024.9	-25020.5	8.79	p < 0.01	8.31	1170E*, 1175S*, 1259S*, 1266Q*, 1267Y**, 1268M*
Cephoidea	Cephidae	*S. parreyssii*	*SMC1*	-23984.2	-23981.5	5.24	p < 0.05	50.11	967S*
			SMC2	-31134.7	-31133.1	3.024	ns	2.30	270G*, 272E *, 888E*
			SMC3	-22275.1	-22275.0	0.125	ns	19.77	-
			SMC4	-33133.7	-33130.0	5.50	p < 0.05	31.20	498K*
			CAPD2	-38824.8	-38811.5	26.595	p < 0.001	42.20	205A*, 1042S*
			CAPD3	-35458.2	-35457.1	2.17	ns	2.67	-
			CAPG	-22504.1	-22497.6	12.951	p < 0.001	49.07	296G*, 331Q*, 670S*
			CAPH	-21344.1	-21337.6	13.096	p < 0.001	86.31	-
			CAPH2	-18665.5	-18662.7	5.491	p < 0.05	38.83	-
			RAD21	-16260.1	-16255.9	8.29	p < 0.005	82.85	248D*, 249E*, 251P*
			SA	-25080.8	-25080.0	1.56	ns	3.60	-
Orussoidea	Orussidae	*O. abietinus*	*SMC1*	-23986.5	-23987.1	0	ns	28.71	-
			SMC2	-31111.6	-31111.4	0.489	ns	1.27	384S*, 426S*, 478L*, 535K*, 893R*, 1166S*
			SMC3	-22272.7	-22271.7	2.016	ns	30.51	-
			SMC4	-33112.1	-33101.2	21.65	p < 0.05	35.79	639S* 809S*, 1123P*, 1146S*, 1257S*, 1420P*
			CAPD2	-38816.3	-38816.3	0	ns	1.00	-
			CAPD3	-35452.7	-35447.1	11.183	p < 0.001	38.94	-

			CAPG	-22512.1	-22511.9	0.373	ns	3.43	-
			CAPG2	-18264.1	-18263.1	21.133	ns	3.51	-
			CAPH	-21331.1	-21331.0	0.078	ns	1.15	-
			CAPH2	-18660.8	-18660.1	1.727	ns	2.10	-
			RAD21	-16260.4	-16258.2	4.35	$p < 0.05$	51.90	555S*
			SA	-25075.3	-25073.4	3.79	ns	27.70	871V*, 1211S*
